# Physiological features of parvalbumin-expressing GABAergic interneurons contributing to high-frequency oscillations in the cerebral cortex

**DOI:** 10.1016/j.crneur.2023.100121

**Published:** 2023-12-16

**Authors:** Katarina D. Milicevic, Brianna L. Barbeau, Darko D. Lovic, Aayushi A. Patel, Violetta O. Ivanova, Srdjan D. Antic

**Affiliations:** a University of Connecticut Health, School of Medicine, Institute for Systems Genomics, Farmington, CT, 06030, USA; b University of Belgrade, Faculty of Biology, Center for Laser Microscopy, Belgrade, 11000, Serbia

**Keywords:** Gamma oscillations, Axon initial segment, Electrical synapse, Myelinated axon, Dendritic integration, GEVI

## Abstract

Parvalbumin-expressing (PV+) inhibitory interneurons drive gamma oscillations (30–80 Hz), which underlie higher cognitive functions. In this review, we discuss two groups/aspects of fundamental properties of PV+ interneurons. In the first group (dubbed *Before Axon*), we list properties representing optimal synaptic integration in PV+ interneurons designed to support fast oscillations. For example: [i] Information can neither enter nor leave the neocortex without the engagement of fast PV+ -mediated inhibition; [ii] Voltage responses in PV+ interneuron dendrites integrate linearly to reduce impact of the fluctuations in the afferent drive; and [iii] Reversed somatodendritic Rm gradient accelerates the time courses of synaptic potentials arriving at the soma. In the second group (dubbed *After Axon*), we list morphological and biophysical properties responsible for (a) short synaptic delays, and (b) efficient postsynaptic outcomes. For example: [i] Fast-spiking ability that allows PV+ interneurons to outpace other cortical neurons (pyramidal neurons). [ii] Myelinated axon (which is only found in the PV+ subclass of interneurons) to secure fast-spiking at the initial axon segment; and [iii] Inhibitory autapses – autoinhibition, which assures brief biphasic voltage transients and supports postinhibitory rebounds. Recent advent of scientific tools, such as viral strategies to target PV cells and the ability to monitor PV cells via in vivo imaging during behavior, will aid in defining the role of PV cells in the CNS. Given the link between PV+ interneurons and cognition, in the future, it would be useful to carry out physiological recordings in the PV+ cell type selectively and characterize if and how psychiatric and neurological diseases affect initiation and propagation of electrical signals in this cortical sub-circuit. Voltage imaging may allow fast recordings of electrical signals from many PV+ interneurons simultaneously.

## Highlights

• PV+ interneurons intercept and filter cortical input, and then intercept and filter cortical outputs. Information can neither enter nor leave the neocortex without strong engagement of fast PV + cell-mediated inhibition..
• Predominantly linear voltage responses in dendrites of PV + interneurons are used to reduce impact of the fluctuations in the afferent drive, which in turn promotes PV + interneuron network synchrony.
• PV + interneurons use fast spiking to outpace other excitable cells in the cortex and sculpt the ongoing electrical rhythm.
• Electrical synapses (void of synaptic delays and high metabolic demands) are formed between PV + interneurons to allow the PV + interneuronal network to multiply its strength, outpace, and overcome other competing networks in the cortex. We envision the cerebral cortex as consisting of two competing cortical networks: one constituted by slow and numerous pyramidal cells releasing glutamate, and the other network composed of fast yet scarce interneurons releasing GABA.
• The vast majority of cortical excitatory pyramidal cells have myelin wraps around their axons. However, among the cortical inhibitory interneurons, only the PV + subclass has myelinated axons, which are used to secure fast spiking in the initial axon segment (first), and then shorten time delays between the PV + interneuron action potential and synaptic releases of GABA at the PV + axon terminals (second).

## Graphical abstract

**Figure:**
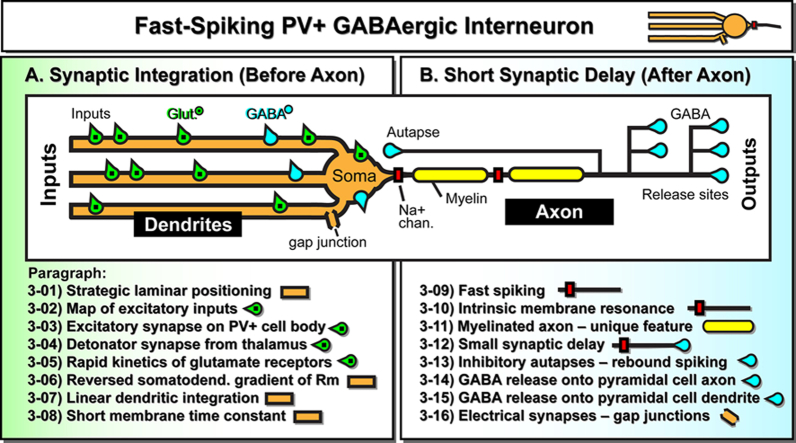

## Introduction

This review centers on the examination of fast-spiking parvalbumin-expressing (PV+) interneurons within the neocortex. Acknowledging occasional limitations in direct neocortical measurements, we have incorporated certain features observed in hippocampal PV+ interneurons (e.g., Reversed Rm gradient, section 3.6). We have assembled a list of interesting anatomical and physiological features that uniquely congregate in one cell type (PV+ interneuron). Some of these features can be found in other neurons (e.g. excitatory input on the cell body, smooth dendrite, fast-spiking, myelinated axon, GABA release), but only the PV+ interneuron class holds a *full set* (Box-1 & Box-2) that makes it *act and work* both, really fast and mighty strong.

Box 1 **Fundamental properties of PV+ interneurons – Before Axon.**

A. Synaptic Integration Before Axon.

3.1) Strategic laminar positioning.
3.2) Map of excitatory inputs.
3.3) Excitatory synapse on PV+ cell body.
3.4) Detonator synapse from thalamus.
3.5) Rapid kinetics of glutamate receptors.
3.6) Reversed somatodendritic gradient of Rm.
3.7) Linear dendritic integration.
3.8) Short membrane time constant.

Alt-text: Box 1

Box 2 **Fundamental properties of PV+ interneurons – After Axon.**

B. Synaptic Delay After Axon.

3.9) Fast-spiking.
3.10) Intrinsic membrane resonance.
3.11) Myelinated axon – unique feature.
3.12) Small synaptic delay.
3.13) Inhibitory autapses – rebound spiking.
3.14) GABA release onto pyramidal cell axon.
3.15) GABA release onto pyramidal cell dendrite.
3.16) Electrical synapses – gap junctions.

Alt-text: Box 2

Gamma-aminobutyric acid (GABA) functions as the principal inhibitory neurotransmitter in the cerebral cortex. Upon binding to GABA-A receptors, GABA elicits either hyperpolarizing or shunting inhibition. Hyperpolarizing inhibition occurs when the equilibrium potential of the GABA-A receptor (E_Cl_) is more negative than the neuron's membrane potential (V_m_), resulting in typical inhibitory postsynaptic potentials (IPSPs). Shunting inhibition, on the other hand, takes place when E_Cl_ equals V_m_ (Burman et al., 2023). Note that transmembrane current (**I**_GABA_) is the product of receptor conductance (g_GABA_) and the driving force (V_m_ – E_Cl_); [ **I**_GABA_ = **g**_GABA_ * (**V**_m_ – **E**_Cl_) ]. In instances where V_m_ equals E_Cl_, an increase in gGABA-A (GABAergic input) may transpire without an overt change in the postsynaptic neuron's membrane potential, as the driving force for the GABA-A current at that moment is zero (V_m_ – E_Cl_ = 0). Both forms of GABAergic signaling, hyperpolarizing and shunting, exert potent inhibition (Burman et al., 2023).

**Fig. 1:**
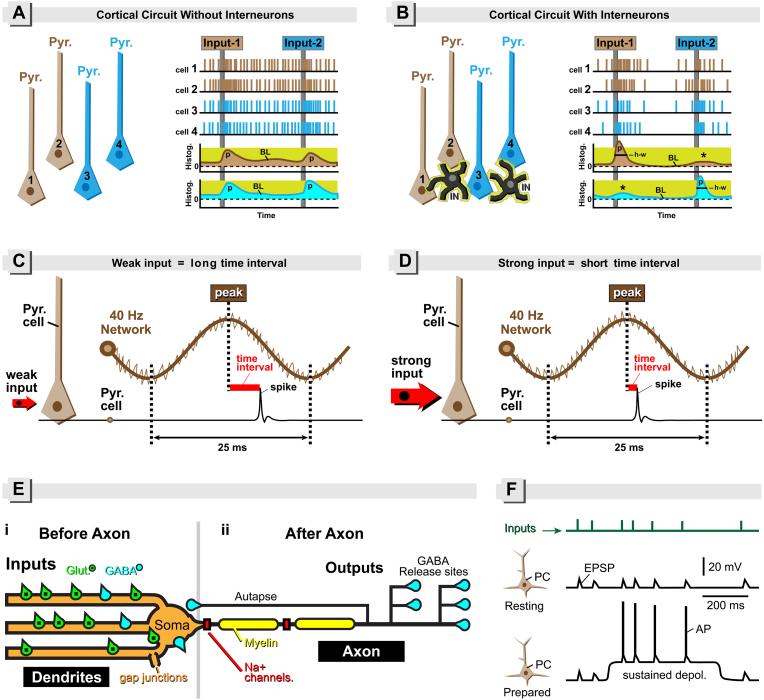
Panels A&B: PV+ interneurons eliminate unnecessary spikes in CNS neurons. (**A**) Left. Cortical circuit void of interneurons (INs). Pyramidal cells “1” and “2” belonging to a “*beige cortical circuit*” are tuned to “*beige inputs (Input-1)*”. The preferred input is Input-1. Pyramidal cells “3” and “4” of the “*blue circuit*” are better tuned to “*blue inputs (Input-2)*”. The preferred input is Input-2. On the right, we have 4 “recordings” from 4 pyramidal cells (Cells 1–4). Each vertical tack marks an AP. Timing of cortical input is marked by long vertical line transecting 4 recording traces. All cells respond to all inputs, except the beige cells “1” and “2” are better tuned to the beige inputs and respond slightly stronger to beige inputs, while blue cells 3 and 4 respond slightly stronger to blue inputs. At the bottom, we have two activity histograms (AP frequency versus time). The beige (upper) histogram plots the average activity of the beige network. The blue (bottom) histogram represents average activity in the blue network of pyramidal cells. Note that both networks (beige and blue) produce small activity peaks time-aligned with cortical inputs. These activity peaks (p) are of a relatively small amplitude (compared to baseline activity). They have very broad shapes, and the baseline electrical activity between two peaks is relatively high (BL). (**B**) The same network as in *A*, except inhibitory interneurons (IN) are “added”. In the presence of interneurons, the total number of APs (spikes) in the PCs (Cells 1–4) has dropped down markedly (compare *A* vs *B*). The baseline activity (BL) is near zero (“sparse activity”). The input-induced peaks are relatively tall and narrow (short half-width) because: [i] the baseline is low; [ii] the rise phase of the activity histogram is faster; and [iii] the decay phase is also faster. Asterisks mark weak responses to non-preferred stimuli. **Panels C&D: Phase coding.** (**C**) Sinusoidal thick brown line represents a cortical network oscillation. Time interval between two throughs is ∼25 ms, hence the frequency of the oscillation is ∼40 Hz. At the bottom, the thin black line is pyramidal cell's membrane potential. Subthreshold depolarizations (EPSPs) are omitted for clarity. A pyramidal cell, PC (*Pyr. cell*) receiving weak synaptic input (red arrow) will generate an AP (*spike*) at some time interval (*time interval*) after the peak (*peak*) of the oscillation occurred. (**D**) If synaptic input into the PC becomes “strong” (red arrow), then the time interval between the one phase of oscillation (e.g. *peak*) and the PC spike (*spike*), is significantly shorter compared to *C*. In this way, the strength of synaptic input into PC is coded by the time interval (thick red line). **(E) Fundamental properties of PV**+ **interneurons.** Combine this panel with text provided in text (Box-1. Before Axon, and Box-2. After Axon). (**E-i**) PV+ interneuron cell body (soma) with smooth (spineless) dendrites receiving excitatory (green) and inhibitory (blue) inputs. (**E-ii**) Axon of a PV+ interneuron equipped with: [i] axonal collaterals, [ii] myelinated internodes, [iii] “supercritical density” of Na+ channels in the axon initial segment & nodes of Ranvier, [iv] multiple GABA release sites including [v] the self-synapses (autapses). **(F) “Prepared state” of a pyramidal neuron**. The same pyramidal cell (PC) responding to synaptic inputs (green raster) is shown in a “*Resting*” state and in the “*Prepared*” state, subsequently. Synaptic inputs can trigger APs in the “*Prepared*” state only. (For interpretation of the references to color in this figure legend, the reader is referred to the Web version of this article.)

GABAergic interneurons comprise 15–20% of all cortical neurons (Beaulieu and Somogyi, 1990). PV+ interneurons comprise 30–40% of the GABAergic interneuron pool, making them the most abundant sub-class of cortical inhibitory interneurons (Bodor et al., 2005; Gonchar and Burkhalter, 1999) – for every 8–10 excitatory pyramidal cells (PCs), there is only one PV+ inhibitory interneuron. How can an outnumbered PV+ interneuron *push* a numerous group of *large and slow* pyramidal cells (PCs), and efficiently drive them into fast electric oscillations, *at a moment's notice*? Perhaps some of the anatomical and biophysical properties listed in previous reviews (Connors, 2017; DeFelipe et al., 2013; Hu et al., 2014; Isaacson and Scanziani, 2011; Jonas et al., 2004; Markram et al., 2004; Whittington et al., 2018; Wingert and Sorg, 2021), and properties which did not receive much attention in PV+ -interneuron review-articles (e.g., interneuron type with a myelinated axon; myelination defects of PV+ cells, resurgent current in the Kv3 channel; linear dendritic integration; type-2-firing; tunable synaptic delays; etc.), can begin to answer this question. By combining text and simple schematic figures, here we explain the most basic electrophysiological concepts [e.g., [▪] Temporal code – phase coding (Fig. 1CD); [▪] Local (dendritic) input resistance (Fig. 3A); [▪] The effect of the membrane time constant (tau) on voltage transients (Fig. 3D); [▪] Synchronized afferent input (Fig. 4B); [▪] Neural network dynamic range (Fig. 4C); [▪] Neuron's resonant frequency (Fig. 4D); etc.].

## 1 Unnecessary spikes

Whether the input is coming locally from neighboring cortical cells, or remotely from a distant brain region (projection fibers), PV+ interneurons will inhibit a *large proportion of the local population* (Wingert and Sorg, 2021). In somatosensory and motor cortices, a few interneuron spikes prevent many unnecessary pyramidal cell spikes (Fries et al., 2007), promoting sparse coding (Pala and Petersen, 2015). We hypothesize that in the absence of intact inhibitory interneuron functions, PCs responding to regular sensory inputs, tend to generate (too) many spikes (Fig. 1A). In contrast, when the inhibitory circuit is intact, PCs generate significantly fewer spikes (Fig. 1B). Rapid and powerful inhibition from PV+ interneurons keeps cortical PCs in a *sparse coding mode*. PV+ cells are necessary to decrease the background basal-level firing (Fig. 1A, BL), allowing PC-population responses to emerge above the background, and to rise fast and fall fast, so there is less activity overlap between two subsequent stimuli in the PC-network. Given that PV+ cells make inhibitory contacts with both pyramidal- and nonpyramidal neurons (with exception of chandelier cells which exclusively innervate PCs and do not establish synapses on other cell types), the simultaneous firing of several PV+ cells could coordinate the activity of the entire cortical network (Atallah et al., 2012; Karnani et al., 2014; Salkoff et al., 2015; Whittington et al., 2018).

As a result of the PV+ cells’ actions, the pyramidal network firing histograms are: [i] sharper (shorter duration (half-width)), [ii] more distinct, [iii] with a better signal-to-noise ratio (SNR), [iv] more meaningful, and [v] with larger dynamic range – network firing does not saturate quickly (Pouille and Scanziani, 2001; Wehr and Zador, 2003). Neuronal activity is “meaningful” if cortical spiking frequency is enhanced for correct (preferred) inputs but suppressed for incorrect (non-preferred) inputs (Fig. 1AB). When sensory inputs last for longer times (e.g. seconds) and actions upon that sensory information are energetically costly, the cortical circuit switches to iterative, *periodic* changes in neuronal activity instead of *continuous* neural activity. The PV+ interneuron actions may thus serve to reduce and economize the information content of neuronal representations (Whittington et al., 2018).

*Feedforward inhibition* is the most basic cortical circuit mechanism, which critically depends on the intact function of PV+ interneurons (Tremblay et al., 2016). In feedforward inhibition, an external source (such as thalamus or neighboring cortex) makes excitatory synapses onto both local PCs and PV+ INs (Buzsaki, 1984). The PV+ INs respond faster than PCs, thus allowing a PV+ IN to inhibit many PC neighbors (Swadlow et al., 1998). Feedforward inhibition is thus a circuit mechanism designed to restrict activity to a small number of cortical PCs, depending on their specialization (e.g. receptive field, temporal window of opportunity) (Isaacson and Scanziani, 2011; Swadlow, 2003; Tremblay et al., 2016). *Lateral inhibition* via PV+ INs (most likely) allows the pyramidal neuron subgroups (e.g., beige subgroup and blue subgroup in Fig. 1B) to respond more strongly to their preferred stimuli (beige input (Input-1) and blue input (Input-2)), and at the same time to have their electrical response to non-preferred stimuli dampened (Fig. 1B, asterisk). In contrast to the specific cross-inhibition (aimed at suppressing a specific sub-circuit, or specific PCs), there could also be a highly divergent broadcast of inhibitory drive from one PV+ cell; everyone is affected within a certain radius of ∼200 μm. Such *blanket inhibition* reduces cumbersome baseline firing (Fig. 1A, BL). An increased sparsity of pyramidal neuron spikes (lower baseline activity) implies less overlap between active subgroups of cortical PCs (Fig. 1B), hence a sharper and more meaningful cortical response. During learning, PV+ interneurons become recruited into stimulus-specific ensembles and provide more selective inhibition as the network becomes better at discriminating behaviorally relevant stimuli (Khan et al., 2018).

An optogenetic study in the visual cortex suggested that without PV+ interneuron activity, the neural response to dissimilar inputs, such as visual objects, becomes more similar and harder to accurately decode (Zhu et al., 2015). PV+ cell activity is needed to reduce the overlap of PC activity in response to two different sensory inputs (Fig. 1AB). Several studies highlight the role of PV+ interneuron-mediated inhibition, which allows for circuits to separate inputs and thus promote the successful encoding and memory retrieval of different stimuli (reviewed in (Wingert and Sorg, 2021)).

*Suppression of the baseline firing (BL) – blanket inhibition.* In somatosensory or frontal rodent cortex, through a widespread and non-specific inhibition of nearly every pyramidal neuron within a 200 μm radius (Karnani et al., 2014; Packer and Yuste, 2011), PV+ cells diminish the activity of PCs without altering their: tuning, sensitivity, class or object preference, and other specific response properties (Atallah et al., 2012; Spanne and Jorntell, 2015; Trachtenberg, 2015).

Pyramidal neuron network dynamic range. When an inhibitory IN subnet is intact, a gradual increase in the intensity of synaptic stimulation produces a very gradual increase in the number of spiking pyramidal cells in the mouse barrel cortex (Fig. 4C, thick green line). All levels of the input strength from 0% to 100% are represented on the input-output curve (thick green line). However, in the absence of inhibitory IN function (for example, in brain slices treated with GABA-A receptor antagonists, picrotoxin or gabazine), a gradually increasing extracellular synaptic stimulation very quickly (Fig. 4C, dashed red line) brings all pyramidal cells to action potential firing (100% of cells are activated at only 20% of the input strength). In the absence of IN function, input strengths greater than 20% are not represented well by the number of activated cells, because the neuronal recruitment is already maximal (saturated) (Fig. 4C). In summary, in the absence of inhibition, the dynamic range of the cortical circuit is ∼20% of what it used to be in the presence of inhibition (Isaacson and Scanziani, 2011).

*Suppression of the out-of-context cells – context inhibition.* The mammalian neocortex processes and stores information using neuronal ensembles. A neuronal ensemble is a dynamic structure composed of synchronously activated neurons engaged in the same task (Eichenbaum, 1993; Engel and Singer, 2001; Hebb, 1949). The same pyramidal cell (PC) participates meaningfully in the function of many different neuronal ensembles (Desimone et al., 1984; Legenstein and Maass, 2017; Wilson and McNaughton, 1993). The same is true for inhibitory PV cells (Lagler et al., 2016). This “time-sharing” feature of the ensemble-organization principle assures a very high number of neuronal ensembles in the CNS that can be assigned to a very high number of specific objects – just as the pixel on a TV screen is only a tiny piece of an image and will be lit up for many other images as well. Here we posit that PV cells play an important role in determining which PC is recruited into a functional neuronal ensemble, and which PC is removed from the ensemble. Through an experience-driven, specific (targeted) inhibition, PV cells may diminish the activity of PCs that should not belong to the ongoing neural ensemble (Cardin et al., 2007; Hirsch et al., 2003). For example, PV+ cells sharply tuned to 135 deg (visual drifting bar) are expected to strongly suppress PCs that are trained to respond to 225 deg (Nowak et al., 2008). We hypothesize that pyramidal cells (PCs) that respond to animal-like objects appearing in the perceptual space, should not respond when plant-like objects appear. We think that UP-state-like depolarizations of the pyramidal neuron's cell body (duration ∼500 ms, amplitude ∼20 mV, Fig. 1F, *sustained depolarization*) play a role in differentiating between wide categories of objects (animal vs. plant). We hypothesize that these long-lasting cell-body depolarizations arise from clustered glutamatergic inputs (inputs on one dendritic branch) which trigger dendritic plateau potentials (Antic et al., 2010). The clustered inputs signal a wide category of objects (e.g., animal-like). Clustered inputs prime (prepare) cortical circuits by bringing a large group of the *animal category-neurons* (PCs that respond to animals) into a sustained depolarized (prepared) state, closer to the action potential (AP) firing threshold (Antic et al., 2018). From this population of depolarized neurons that code one relatively large object class (e.g., animal-like objects), additional synaptic inputs (Fig. 1F, *Inputs*) begin to impinge onto the 20-mV depolarized (prepared) neurons. These additional inputs, distributed across an entire dendritic tree, code specific features of an actual animal species (e.g., four-legged, large, black, dangerous, etc.) and they manage to recruit a very specific ensemble of cells into a *group firing mode*. A well-defined (very specific) ensemble (group) of the AP-firing CNS-cells is always used to code for black bear, regardless of whether a black bear appears in the visual field or gets recalled from memory. We hypothesize that the same group of neurons is active (generate APs) when a black bear appears in the visual field, but also when black bear appears in our thoughts. In this way, we do not need to invent two algorithms: one for perceiving, and the other one for thinking about. Instead, the same algorithm is used for detecting, perceiving, memorizing, and recalling one specific item of information (Antic et al., 2018).

Why is a sustained depolarization (∼20 mV amplitude) of neocortical PCs needed? Because it would be difficult to activate any given specific set of PCs (functional neural ensemble) starting with “cold” PCs that are dwelling at their resting membrane potentials (−70 mV) (Fig. 1F, *Resting*). The voltage threshold for spike firing in a PC axon is > 20 mV above the PC resting potential, and excitatory postsynaptic potentials (EPSPs) arriving on distal dendritic segments strongly attenuate on the way to the cell body. These distal EPSPs generate miniature, sub-millivolt depolarizations of the PC axon (Fig. 1F, EPSP) (Williams and Stuart, 2003). Therefore, it is necessary to keep PCs in depolarized states (prepared states, 20 mV above resting, Fig. 1F, *Prepared*), close to the AP firing threshold (>20 mV above resting), to give the valuable EPSPs a chance to activate the axon. Note that during the *prepared state* (UP state), cortical PCs receive strong barrages of both excitatory and inhibitory postsynaptic potentials, with the inhibitory potentials showing much higher power at all frequencies above 10 Hz and more synchrony between nearby neurons (Hasenstaub et al., 2005; Salkoff et al., 2015). Firing patterns of PV+ basket cells might support and temporally organize neuronal ensembles of the neocortex during various brain states and tasks, including non-REM sleep and working memory-guided decision making (Funk et al., 2017; Lagler et al., 2016).

## 2 Fast electrical oscillation - gamma band – temporal code

Neuronal gamma-band synchronization (30–80 Hz) appears to be a fundamental mode of neuronal activity (Fries et al., 2007; Jefferys et al., 1996; Lasztoczi and Klausberger, 2017; Salkoff et al., 2015). High-frequency oscillations, if defined in terms of the local field potential (LFP), emerge from the summed extracellular currents flowing across the membranes of all cells, not just the PV cells (Buzsaki et al., 2012). However, PV+ interneurons are the main component of the cortical circuitry that generates fast cortical oscillations (Cardin et al., 2009; Dubey et al., 2022; Sohal et al., 2009; Traub et al., 1996).

Based on undergoing electrical gamma oscillation, the amplitude of excitatory input to a PC is converted into a *temporal code*. That is, the amplitude of excitation is represented by the *time delay* between the output spikes relative to the gamma cycle peak; stronger inputs leading to earlier responses (Fries et al., 2007; McLelland and Paulsen, 2009). Pyramidal cells driven weakly will fire late in the oscillation cycle (Fig. 1C, *time interval* is long), whereas those driven strongly will fire earlier (Fig. 1D, *time interval* is short). Such *phase coding* is a version of *temporal coding* often used by CNS neurons involved in rapid processing of a sensory scene (Gerstner, 2021). The brain may use phase-coding to segregate related from unrelated aspects in the perceptual scene. In theory, only one oscillation cycle is needed for the PC to signal the strength of its input to other members of the cortical network. PV+ interneurons are thought to maintain the neocortical reference clock for phase coding*,* and they keep the duration of the oscillation cycles short. For example, consider ongoing network oscillations at ∼40 Hz, where each cycle lasts only 25 ms. Based on the phase-coding theory, the brain takes only 25 ms to find out the input strength received by each PC involved in the active network (neural ensemble). According to Resulaj et al. the brain may require 40–80 ms (Resulaj et al., 2018), but nevertheless, the point here is that just one or two oscillation cycles are enough. PCs which did not reach depolarizations required to generate axonal spikes, did not deliver any signals to their postsynaptic targets, did not speak their voice, hence they are invisible to the conscious brain.

Every great simple rule (*the GAMMA band (30–*80 Hz*) is essential for phase coding*) has a twist. In rat hippocampus brain slices, using whole-cell recordings and simulated oscillations at 5 Hz or 40 Hz, researchers found that during the *THETA band* (5 Hz) frequency oscillations, the phase of the first spike per cycle was a near-linear function of tonic excitation, advancing through a full 180 deg, from the peak to the trough of the oscillation cycle as excitation increased. In contrast, this relationship was not apparent for *the GAMMA band* (40 Hz) oscillations, during which the phase of neuronal firing was independent of the level of tonic excitatory input (McLelland and Paulsen, 2009).

## 3 Fundamental properties of fast-spiking PV+ inhibitory interneurons

In this review, properties of PV+ interneurons that are essential for the production of fast oscillations are segregated into two lists, based on their site of action. If a selected property largely affects integration of electrical signals in dendrite and PV+ soma, before triggering of axonal AP, then it is listed in group A: *Before Axon* (Fig. 1E–i). If, on the other hand, a selected property affects physiological events occurring after an AP had already been initiated in the PV+ axon, then this property is listed in group B: *After Axon* (Fig. 1E–ii). Traditional terms for “Before Axon” could be “Afferent Processes” or “Input Pathways,” encompassing aspects such as synaptic integration. On the other hand, “After Axon” could be described as “Efferent Processes” or “Output Pathways,” involving activities like neurotransmission. These terms align with conventional terminology used in neuroscience to distinguish between the incoming and outgoing signals of a neuron.  

A. Before Axon

In cortical PV+ interneurons, the synaptic integration process has evolved to assure sensitive responses to incoming excitatory inputs (e.g. thalamocortical projections; cortico-cortical projections), and efficient transfer of fast synaptic rhythms into the PV+ interneuron output (triggering of APs in the PV+ axon).

### 3.1 Strategic laminar locations to control both cortical input and output

**Fig. 2:**
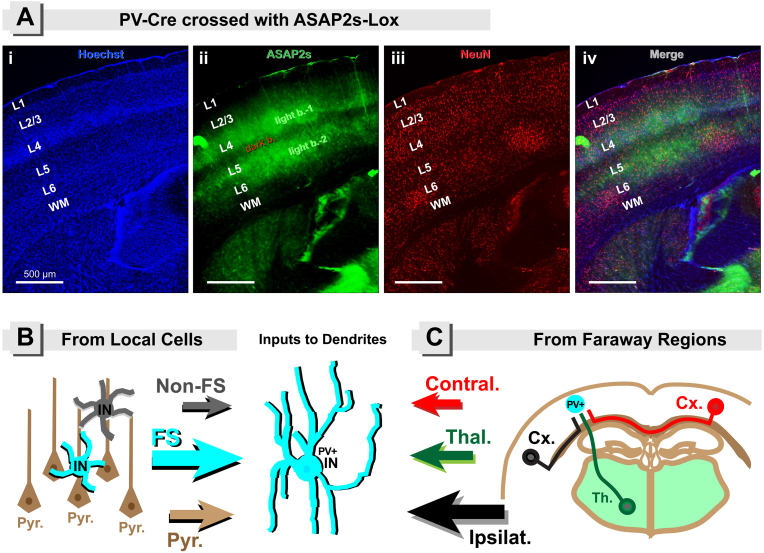
Panel A: Laminar positioning of PV-positive interneurons. (**A-i**) Brain section (50 μm thickness) cut from a PV-ASAP2s mouse. Unpublished data. Nuclear stain (Hoechst) reveals a high-density granular layer – cortical layer 4 (L4). (**A-ii**) Genetically encoded fluorescent membrane protein (ASAP2s) is in the PV+ interneuron (IN) plasma membrane, including cell body, dendrites, and axons. Dendrites and axons of the ASAP2s-labeled PV+ INs form two bright bands (*light b.-1* & *light b.-2*). The dark band (dark b.) corresponds to the upper portion of the cortical layer L5. Note how green neurites of PV+ INs decline (stop) at the border between L2 and L1; and they also decline in the section of white matter (WM) immediately attached to striatum. (**A-iii**) Nuclei of the post-mitotic neurons (all neurons including PCs and INs) are labeled with Anti-NeuN antibody. (**A-iv**) Merge of three channels A-C, reveals two bright bands of PV+ neurites. The upper PV+ band is in layers L2/3/4, while the lower PV+ band is in layers L5b/6. **Panels B&C: Afferent input to PV+** **interneurons.** In the center, we have a fast-spiking (FS) PV+ cortical inhibitory interneuron (turquoise). This cell is postsynaptic in our scheme – it will receive inputs from the *Panel B* (local) and from *Panel C* (remote). (**B**) Local neurons (same cortical column) provide afferent synaptic innervation to dendrites of PV+ IN. These local presynaptic neurons include excitatory PCs (brown cells with apical dendrites) and two classes of inhibitory interneurons (FS and Non-FS). Note that presynaptic FS interneurons make strong GABAergic synapses in the proximal dendritic segments, cell body and proximal axon of the postsynaptic PV+ cell. PCs uniformly form appositions with PV+ interneurons in all layers, except layer 6 (L6). The L6 PCs (*corticothalamic projections*) form appositions to PV+ cells at a significantly higher rate than other PCs (Kuramoto et al., 2022). (**C**) PCs in the ipsilateral cortex (black), PCs in the contralateral cortex (red), and thalamic neurons (green), supply afferent innervation to dendrites of the postsynaptic PV+ cell. Most of the PV+ cells within and near layer 4 (L4), and some of FS cells in layer 5 (L5), receive a potent monosynaptic input from thalamus (Swadlow et al., 1998). Projections from S2 to non-whisker S1 (which are important for coordinated paw movement) preferentially innervate PV inhibitory interneurons (Chang et al., 2022). (For interpretation of the references to color in this figure legend, the reader is referred to the Web version of this article.)

Unlike pyramidal neurons which are born in the subventricular zone of developing cortex, the GABAergic PV+ interneurons are born outside of cortex, in the medial ganglionic eminence. After migrating to the cortex (Butt et al., 2005; Xu et al., 2004), PV+ interneurons assume their final positions in the neocortex, with roughly two major horizontal bands (Fig. 2A–iv). The upper band of PV+ interneurons covers layers 2–4 (Fig. 2A–ii, *light band-1*). L4 contains cells which receive massive sensory inputs from thalamus. Layers (L2 and L3) contain pyramidal neurons that make cortico-cortical projections – communication between brain areas of different hierarchy (e.g., primary, secondary, association), or different modality (e.g., motor, visual, auditory). The lower horizontal band of PV+ interneurons (*light band-2*) occupies putative L5b (Fig. 2A–i & iii), which contains pyramidal neurons that project outside cortex – major communication between cortex and spinal cord (pons, superior colliculus, etc.). The dark band between two light bands is aligned with the upper portion of L5 (putative L5a). In both rodents and primates, PV+ interneurons are most densely concentrated in a horizontal band centered on L4 (Williams et al., 1992) (Fig. 2A, L4).

***Key point***. *Information can neither enter nor leave the neocortex without the engagement of the fast PV+ -mediated cortical inhibition. Cortical PV+ interneurons shorten and sculpt cortical inputs as they arrive in cortex, and at the same time they shorten and restrain cortical outputs as they leave cortex. Evolutionary newer brain regions (e.g. cortex), just like modern people (e.g. users of social media platforms), prefer brief and straight to the point messages.*

**Fig. 3:**
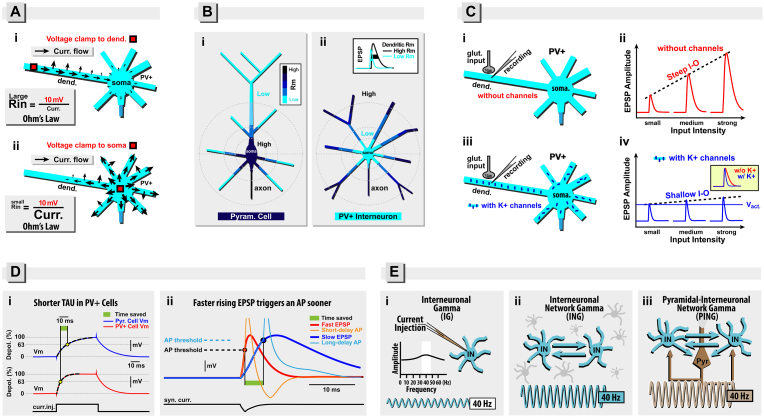
Gradient of specific membrane resistance (Rm). (**A-i**) Schematic drawing of a dendrite (dend.) attached to the cell body (soma) of a CNS neuron. A voltage step (10 mV) is generated through an electrode inserted into distal dendrite (red rectangle). During a voltage clamp step of 10 mV, current leaks out through holes in the plasma membrane, and flows toward ground electrode. The extracellular space surrounding CNS neurons is equipotential. The flow of current toward cell body (soma) is reduced by high axial resistance of the dendrite (dend.). The total current (**Curr.**) in this electrical circuit is proportional to the sum of all black arrows. In this example (dendritic voltage clamp), **Curr.** is relatively small, hence input resistance (Rin) is large (Ohm's Law). (**A-ii**) The same CNS neuron, except a voltage-clamp electrode (red rectangle) is now inserted into the cell body (soma). Due to a low somatic axial resistance, and due to a large membrane area contained in the perisomatic membrane (cell body and proximal dendrites), there are many membrane pores through which large currents can leak out. The total current (**Curr.**), proportional to the sum of all black arrows, is relatively large, hence Rin is small (Ohm's Law). (**B-i**) In PC, the specific membrane resistance (Rm) decreases with the distance from the cell body. Light blue indicates low Rm. Dark blue and black indicate high Rm. (**B-ii**) In PV+ interneuron, Rm increases with distance from the soma. Inset: An identical glutamatergic input intensity is used to generate two EPSPs in the same distal dendrite, except when local dendritic Rm is high the EPSP amplitude is large, and duration is long (black trace). On the contrary, when local dendritic Rm is low, the EPSP amplitude is small, and duration is short (light blue trace). (**C-i**) A glutamatergic input impinges onto a dendrite of PV+ interneuron. The resulting EPSP is recorded at the input site (in dendrite) and displayed in *C-ii*. (**C-ii**) In PV+ interneuron lacking voltage-sensitive channels, a gradual increase in glut. input intensity results in a gradual increase in EPSP amplitude. The I–O function (dashed black line) has a steep slope (e.g. high gain). (**C-iii**) The same PV+ interneuron as in *C-i*, except “*high-threshold voltage-gated K* + *channels”* are now functional in the plasma membrane. (**C-iv**) In PV+ interneuron with functional K+ channels, the I–O function (dashed black line) has a shallow slope (e.g. low gain). The blue horizontal line marks the voltage level at which the K+ current activates strongly (**V**_**act.**_). **Panel D: Membrane time constant (tau).** (**D-i**) Current injection pulse (curr. inj.) produces characteristic transient depolarizations in two injected neurons (blue trace from PC and red trace from PV+ interneuron). Exponential fit through the charging curve (dashed black line) is used to estimate tau. Yellow ball marks the amount of time needed to reach 63% of the maximal depolarization (steady state, 100%). Due to a slower tau, the “blue” PC (Pyr. Cell) requires approximately three times longer to reach 63% of the steady state, compared to the “red” PV+ interneuron. Green rectangle marks time “saved” due to a shorter tau in PV+ cell. (**D-ii**) The bottom trace marks an excitatory synaptic current entering the neuron. Voltage waveforms of excitatory postsynaptic potentials EPSPs in a pyramidal neuron (blue trace, long tau) and PV+ interneuron (red trace, short tau) are superimposed. The red ball marks an intersection between EPSP and action potential (AP) voltage threshold in the PV+ cell. At this moment (red ball), an AP is initiated in the PV+ cell (orange trace). The pyramidal cell (blue trace) initiates an AP much later. Blue ball marks the time point at which blue EPSP crosses the AP voltage threshold when tau is long. The rising slope of the blue EPSP is so slow that by the time the voltage threshold is reached (blue ball) a significant fraction of voltage-gated sodium channels had been inactivated by depolarization. The density and dynamics of all channels (including Na channels) in two cells (red and blue) are identical. **Panel E: Theoretical models of gamma oscillations.** (**E-i**) According to the *IG Model*, variable or sustained intracellular current injections into fast-spiking cortical interneurons (IN) most often produce rapid voltage transients, or even spikes at ∼40 Hz. The IN intrinsic membrane properties seem to push the IN cell electrical responses to “resonate” at ∼40 Hz (white region in the inset). (**E-ii**) According to the *ING Model*, a neuronal network composed entirely of fast-spiking inhibitory interneurons most often produce fast network oscillations at ∼40 Hz. Adequate chemical and electrical synaptic coupling among INs are sufficient to generate 40 Hz oscillations. (**E-iii**) In this model of gamma frequency oscillations (PING Model), the excitatory pyramidal cells are critical (necessary) components that lead and drive IN responses, resulting in a gamma oscillation encompassing the whole cortical network. (For interpretation of the references to color in this figure legend, the reader is referred to the Web version of this article.)

**Fig. 4:**
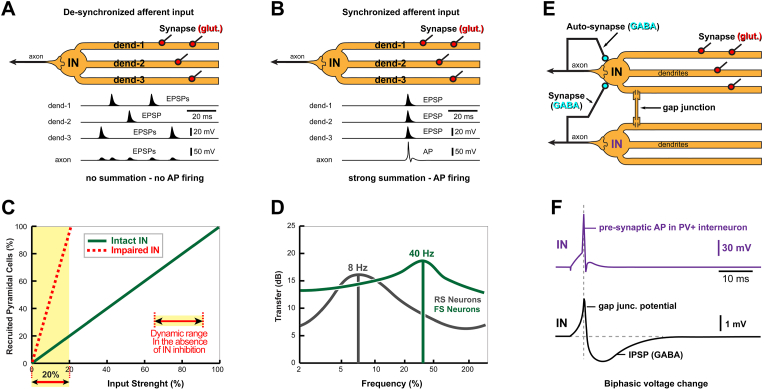
Features and influence of PV+ interneurons. (**A**) EPSPs are tall and sharp at the input site in dendrite, but smaller and slightly broader after they reach the axon (cable filtering). Membrane time constant (tau) is short hence the summation time window is missed – no temporal summation – no action potential firing in the PV+ axon. (**B**) Dendritic EPSPs are now synchronized (occur in a narrow window of time) – this brings the axon above the AP firing threshold. (**C**) When the inhibitory interneuron network is intact, a gradual increase in the intensity of synaptic stimulation produces a very gradual increase in the number of spiking pyramidal cells (thick green line). The dynamic range is full 100%. This means, all levels of the input strength from 0% to 100% are represented on the input-output curve (thick green line). However, in the absence of inhibitory interneuron (IN) function (e.g. in brain tissues treated with GABA-A receptor antagonists picrotoxin or gabazine), extracellular synaptic stimulation quickly (dashed red line) bring all pyramidal cells to AP firing (100% of cells are activated at only 20% of the input strength) (Isaacson and Scanziani, 2011). In other words, in the absence of inhibition (dashed red line), the dynamic range of the cortical circuit is only 20% of what it used to be in the presence of inhibition (thick green line). Input strengths greater than 20 % are not represented well by the number of activated cells (dashed red line). (**D**) Transfer function of the injected-current to spike-times for the RS (pyramidal) and FS (PV+) neurons. When intracellular injection of colored noise is presented to various cortical neurons, the spiking output depends on the cell type. Excitatory pyramidal cells (RS) produce the strongest response to ∼8 Hz stimulation frequency. The fast-spiking (FS) cells, putative inhibitory PV+ interneurons, produce the strongest responses when synaptic stimulation is ∼40 Hz. The transfer function (Transfer) of the FS cells (thick green line) is most efficient in the gamma frequency range (Hasenstaub et al., 2005). (**E**) Two fast-spiking PV+ interneurons communicate through both chemical (GABA) and electrical (gap junction) synapses. Inhibitory chemical synapses are on the cell body and proximal dendrites. Electrical synapses are between dendrites, often in proximal dendritic segments. PV+ interneurons also make autapses (Auto-synapse, GABA). PV+ autapses are made by the axon of a PV+ interneuron on its own dendrites or cell body (Galarreta and Hestrin, 2001; Szegedi et al., 2020). Excitatory glutamatergic synapses (Synapse (glut.)) arise from sources described in Fig. 2 and impinge predominantly on thin dendritic branches void of dendritic spines. (**F**) Action potentials in one PV+ interneuron (purple trace) produces a biphasic response in the neighboring PV+ interneuron (black trace) (Gibson et al., 1999). The initial depolarizing transient is due to current passing through a gap junction, shown in *E*. The subsequent hyperpolarizing component is due to chemical synapse (*Synapse, GABA*) shown in *E*. Note that the peak depolarization (first) and peak hyperpolarization (second) occur one after another within a very short time interval, in the order of 10 ms. (For interpretation of the references to color in this figure legend, the reader is referred to the Web version of this article.)

*Cortical inputs intercepted by the PV+ interneurons.* In the somatosensory cortex (S1), most of the PV+ cells within and near layer 4 (L4), and some of PV+ cells in layer 5 (L5), receive a potent monosynaptic input from ventrobasal (VB) thalamus (Swadlow et al., 1998). Barreloid neurons have axons that diverge strongly to provide synaptic input to nearly all of the fast-spiking interneurons of the aligned S1 barrel column (Swadlow, 1995). Terminal arbors of individual VB thalamocortical afferents virtually fill their corresponding S1 barrel (Jensen and Killackey, 1987), which explains the high level of synchronism among PV+ interneurons belonging to the same whisker barrel. Upon receiving sensory thalamic input, the PV+ interneurons activate (spike) vigorously and inhibit local excitatory neurons strongly, thus creating a robust feedforward inhibitory circuit (Gabernet et al., 2005; Sun et al., 2006) that may serve to: [i] sharpen the postsynaptic responses (Fig. 1B), [ii] widen dynamic range of population responses, and [iii] prevent cortical overexcitation (Bruno and Simons, 2002; Wehr and Zador, 2003). Thalamocortical impulses will generate only a brief ‘*window of excitability*’ during which spikes can occur in the post-synaptic targets of fast-spiking interneurons (Swadlow, 2003). Single pulse stimulation of the hippocampal CA1 or subiculum region induce an excitatory response in 70% of recorded interneurons in the prelimbic and medial-orbital areas of the rat prefrontal cortex (Tierney et al., 2004). Hippocampal projections to prefrontal cortex carry spatial information, and support working memory (Ciocchi et al., 2015; Spellman et al., 2015), where the successful encoding of hippocampal cues appears to be mediated by oscillation-synchrony between hippocampus and PFC (Hartwich et al., 2009; Spellman et al., 2015).

*Cortical outputs allowed to go out by the PV+ interneurons.* The anatomical clustering of PV+ interneurons in the output layers L5/6 and L2/3 (Fig. 2) suggest their important role in shaping the cortical outputs. Superficial layers (L2 and L3) are thicker in primates than in other species (Hutsler and Galuske, 2003) and participate heavily in higher cognitive functions. In large scale models, L2/3 interneurons appeared largely responsible for gamma activation through preferential attenuation of the rest of the spectrum (Neymotin et al., 2011). Large basket cells in L2/3 have relatively long axons contacting excitatory (pyramidal) and PV+ cells (Kisvarday et al., 1993), which may allow a long-range synchrony of the gamma oscillations (Engel et al., 1991a, 1991b; Traub et al., 1996), which supports perceptual grouping (Woelbern et al., 2002); a process of determining which regions and parts of the perceptual scene (visual, auditory, somatosensory) belong together as unitary objects.

### 3.2 Map of the afferent excitatory inputs onto PV+ interneuron dendrites

Within the mouse cerebral cortex, the primary neuronal targets of feedforward and feedback cortico-cortical projections between two hierarchical cortical areas (e.g. primary and secondary sensory area) are PCs and the PV+ interneurons (Gonchar and Burkhalter, 1999). While the abundance of contacts on PC is of no surprise, the abundance of cortico-cortical inputs intercepted by PV+ strongly suggests that this inhibitory interneuron class constitutes feedforward inhibition (Bagnall et al., 2011; Swadlow et al., 1998). For didactic purposes, we divide afferent excitatory inputs to PV+ cells into two groups: *Local* and *Faraway*.

*Local - From Local Cells*. The local group is made of cells in the same cortical column (or the neighboring column) (Dantzker and Callaway, 2000), including inhibitory fast-spiking (FS) PV+ interneurons, inhibitory non-FS interneurons, and excitatory PCs (Fig. 2B).

*Faraway - From Faraway Regions*. Any axon projection originating outside the cortical column (in which our PV interneuron resides) is here denoted “faraway” or “remote”. Remote brain regions supply excitatory glutamatergic inputs onto neocortical PV+ interneurons, and these include: the thalamus, ipsilateral cortex, and contralateral cortex (Fig. 2C). In prefrontal region of neocortex, long range inputs also include amygdala and ventral hippocampus (Yang et al., 2021). The most abundant faraway afferents are from the ipsilateral cortex, and they can roughly be divided into feedforward (from lower to higher processing hierarchy) and feedback (from higher to lower information processing hierarchy). Interestingly the feedforward cortico-cortical contacts onto PV+ interneurons are: [i] significantly larger in diameter, [ii] contain more mitochondria, and [iii] contain more docked synaptic vesicles, compared to the feedback cortico-cortical contacts (Gonchar and Burkhalter, 1999).

In Fig. 2, the size of an arrow suggests the relative number of inputs contributed by that cell class. For example, FS PV+ cells provide the greatest fraction of local afferents onto the PV+ cell dendrites (Fig. 2B, turquoise arrow), and these contacts are inhibitory in nature. Two kinds of *local* cells (cortical L5-L6 pyramidal neurons and L4 stellate cells) and *faraway* cells (thalamic projection neurons) provide excitatory glutamatergic synapses onto PV+ interneurons (Bagnall et al., 2011; Gonchar and Burkhalter, 1999; Komlosi et al., 2012; Molnar et al., 2016; Swadlow et al., 1998). In PV+ L4 basket cells of the cat visual cortex, equal numbers of synapses are provided by the L6 PCs (∼40%) and the spiny stellate cells (∼40%), whereas the *faraway* thalamic afferents contribute only ∼15%. As much as 79% of the inhibitory GABAergic synapses originate from other L4 basket cells (Ahmed et al., 1997). Local spiny stellate and *faraway* thalamic synapses are preferentially located on the soma and proximal dendrites of PV+ interneurons, while *faraway* cortico-cortical projections are rather located on the distal dendrites (Ahmed et al., 1997; Bagnall et al., 2011; Freund et al., 1985).

### 3.3 Afferent excitatory synapse on the PV+ cell body

Cortical pyramidal neurons rarely receive excitatory synaptic inputs on their cell bodies. As a result, in PCs, EPSPs are relatively small in amplitude (Williams and Stuart, 2003) and almost never reach the threshold for triggering action potentials. In contrast, cortical PV+ interneurons receive considerable excitatory synaptic input directly on their cell bodies (Keller and White, 1987; Peters et al., 1991; Wang et al., 2002), which should lead to a potent postsynaptic response (Gao and Goldman-Rakic, 2003; Molnar et al., 2016). Fast-spike GABAergic cells display EPSPs with a rapid rise time (Avermann et al., 2012; Gao and Goldman-Rakic, 2003; Thomson and Deuchars, 1994; Thomson et al., 2002) which suggest an excitatory synapse positioned near or at the cell body (cable theory). *Path distance* between synaptic input site (e.g. dendrite) and recording site (e.g. cell body) influences the shape of the recorded EPSP (Williams and Stuart, 2003). Due to cable filtering, greater path distances produce heavier filtering of the EPSP voltage waveforms, resulting in reduced EPSP amplitude, slower EPSP rising and decay times. Therefore, the advantages of positioning a *pyramidal cell-to-interneuron synapse* directly onto the interneuron-cell body are: [i] large EPSP amplitude; [ii] faster EPSP rising time; and [iii] faster EPSP falling time. The rising slope of EPSP is essential for efficient recruitment of voltage-gated Na^+^ channels. If PC-to-IN synapse is near the interneuron (IN) cell body, then single action potentials in PCs can trigger unitary (EPSPs) that reliably produce an AP in a postsynaptic PV+ interneuron. Importantly, this behavior (unitary EPSP producing an AP) is often seen in PC-to-PV+ connections, but it is not seen in other monosynaptically-connected cortical neurons (Jouhanneau et al., 2018).

In the human cortex, strong multivesicular excitatory synapses may connect L2/3 pyramidal cells to GABAergic interneurons with very large suprathreshold EPSPs (Molnar et al., 2016). A solitary spike in a human PC, triggers firing in the local interneurons, initiating a population burst known as a complex event (Molnar et al., 2008). In summary, PV+ interneuron firing evoked by a solitary PC spike has now been found in both rodents and humans (Avermann et al., 2012; Jouhanneau et al., 2018; Szegedi et al., 2017). Remarkably, the time interval (latency) from the presynaptic PC spike to a monosynaptic EPSP in postsynaptic interneuron can be less than 1 ms. The cell body diameter, total dendritic length, and horizontal and vertical spans of the axonal arbor of PV cells were similar in monkeys and rats, although the monkey basket cells appeared to be more excitable (Gonzalez-Burgos et al., 2005; Povysheva et al., 2008). In rodents, monkeys and humans, the maximal firing frequency of FS neurons increases with the species hierarchy, and varies based on the cortical area and age, with mean firing frequency often exceeding 400 Hz (Wang et al., 2016). The cell body of human neocortical PV+ basket interneuron amply express HCN channels, which shorten the lag between excitatory postsynaptic potentials and action potential generation in human, whereas these channels are sparse at the rodent basket cell soma membrane (Szegedi et al., 2023).

In summary, as a consequence of [a] synapse location (near or directly on the cell body) and [b] synapse strength (multivesicular release), the FS PV+ interneurons receive unitary excitatory postsynaptic potentials with: [i] higher probability, [ii] larger amplitudes, [iii] faster kinetics, and [iv] shorter synaptic latency, compared with PCs or other GABAergic interneurons (Avermann et al., 2012; Pala and Petersen, 2015). This arrangement assures that an FS PV+ neuron is activated each time a neighboring PC engages in firing.

### 3.4 Afferent thalamic input - excitatory synapse – detonator synapse

Activation of just one thalamic afferent can fire interneurons (Cruikshank et al., 2007; Swadlow and Gusev, 2000). This is because an individual thalamic afferent forms multiple (∼15) contacts on PV+ dendrites. These contacts release glutamate with high probability, yielding large conductance change, up to 10 nS. To exert maximal efficacy (assessed by axonal depolarization), thalamic contacts should impinge at or near the cell body (cable theory). Dendritic Ca imaging and electron microscopy showed that thalamic contacts indeed arrive on proximal dendritic branches of PV+ interneurons, preferentially near branch points, and each thalamic bouton can release multiple vesicles simultaneously (Bagnall et al., 2011). In summary, thalamic “detonator” synapses assure robust involvement of L4 and L5 PV+ interneurons in curtailing cortical sensory activity (via feedforward inhibition) for any given sensory barrage arriving from the thalamus. As a result, in sensory cortices, PCs experience EPSP-IPSP sequences; phasic activation (Fig. 1B) instead of tonic activation (Fig. 1A).

### 3.5 Afferent input - rapid kinetics of glutamate receptors

In the cortex and hippocampus, temporal dynamics of AMPA-receptor-mediated excitatory postsynaptic current influences the rise time of an EPSP. It is thought that AMPA receptors on hippocampal FS PV+ cells are made of AMPA subunit isoforms especially adapted to produce fast rising postsynaptic currents (discussed in (Jonas et al., 2004)). Neocortical FS PV+ cells may indeed employ fast-acting AMPA receptors, as their EPSP voltage waveforms are also very rapid – fast rising.

### 3.6 Reversed gradient of the membrane resistance (Rm) along the somatodendritic axis

On average, the input resistances of neocortical fast-spiking (FS) interneurons are slightly higher than in neocortical PCs (Baranyi et al., 1993; Kawaguchi, 1993a). Ohm's Law specifies that voltage is generated when current is passed through a resistor: V=I x R. When input current (I_in_) is delivered into a neuron, the resulting neuronal membrane potential change (dVm) will be proportional to the effective input resistance (R_in_) of that neuron: dVm = I_in_ x R_in_. For the same amount of excitatory input current, a neuron with high input resistance (i.e. FS PV+ interneuron) will become more depolarized than a neuron with low input resistance (i.e. pyramidal cell). Hence, high input resistance helps PV+ interneurons to reach membrane potentials that can cross AP voltage threshold.

In a CNS neuron characterized with uniform specific membrane resistance (Rm) throughout the dendritic tree, the local input resistance (Rin) varies with morphology. Different cellular compartments (e.g. soma, proximal dendrite, distal dendrite, and axon) may have identical Rm but vastly different local Rin. This is because Rin depends on the path of the lowest resistance from the injection site to the ground-wire positioned in the extracellular space (The Late Gordon Murray Shepherd, personal communication). The path to the ground (reference electrode) is not the same for a recording electrode in the distal dendrite (Fig. 3A–i) versus a recording electrode in the cell body (Fig. 3A–ii). The current injected via somatic electrode has a significantly greater number of electrical paths (black arrows) through pores in the neuronal plasma membrane, compared to the current entering via an electrode positioned in distal dendrites. Hence, the local Rin of the soma is lower than the local Rin of the dendrite.

Dual (soma & dendrite) patch electrode recordings combined with computational modeling indicate that specific membrane resistance (Rm) in hippocampal PV+ interneurons is markedly nonuniform, with a 40-fold higher value in the axon, and a 10-fold higher value in the distal dendrites, than in the proximal dendrites and the soma (Norenberg et al., 2010). This property, although likely conserved across brain regions, remains to be empirically ascertained for cortical PV cells. In hippocampus, the PV+ somatodendritic Rm distribution is nonuniform; there is a gradient of Rm, and direction of this gradient is opposite to that found in PCs (Norenberg et al., 2010). In pyramidal cells, Rm decreases from soma to dendrite (Fig. 3B–i), while in PV+ cells, Rm increases from soma to dendrite (Fig. 3B–ii). Rm affects both the amplitude and the temporal dynamics of local voltage waveforms. While keeping all membrane parameters the same (same specific membrane capacitance (Cm), specific axial resistance (Ra), and morphology), one can test high-Rm and low-Rm by biophysical modeling (computational model). With high-Rm, an EPSP has a large amplitude and long duration (Fig. 3B–ii, inset, black trace). With low-Rm, the EPSP amplitude is smaller (due to reduced Rin), and the shape is narrower (Fig. 3B–ii, inset, turquoise trace) due to a shorter membrane time constant). It has been argued that the inverse Rm gradient (low Rm in soma and high Rm in dendrite, Fig. 3B–ii) is a key factor of rapid signaling in PV+, which ensures rapid propagation from dendrite to cell body, and fast termination of EPSPs (Norenberg et al., 2010). The observed PV+ cable properties (inverted Rm gradient) accelerate the time courses of synaptic potentials arriving at the soma and reduce the amount of resistor-capacitor (RC) filtering that each EPSP would encounter if otherwise the somatic and perisomatic Rm were high. Because somatic and perisomatic Rm is actually low (Fig. 3B–ii), the low-pass RC filtering is light, hence the fast rise, and fast termination of EPSPs.

There is a risk associated with generalizing published findings. For instance, the primary assertion of a ‘reversed Rm gradient’ relies on the study by Norenberg et al., in 2010, conducted specifically in the dentate gyrus — a region notably distinct from the neocortex. The dentate gyrus represents a highly specialized area, and it's essential to recognize that the characteristics of PV cells in other cortical areas may differ significantly.

*Active membrane properties shape the EPSPs in PV+ interneurons*. In dendrites of PV+ cells, the Rm is relatively high (Fig. 3B–ii, dark-colored structures). Because the high dendritic Rm would make local dendritic EPSPs large and long (Fig. 3B–ii, inset, black trace), there must be some additional membrane mechanism to shorten the EPSP voltage waveforms and make them slim, as found in somatic recordings from PV+ cells (Thomson and Deuchars, 1994). High-threshold fast-acting K^+^ current seems to be responsible for slimming the EPSPs waveforms (Hu et al., 2014). In addition to shortening the EPSP half-width, these K^+^ channels also compress EPSP peak amplitudes. For example, in PV+ cells void of any voltage-gated conductances, a gradual increase in glutamatergic input arriving on a distal dendritic segment (Fig. 3C–i) would produce gradually increasing local EPSPs (Fig. 3C–ii). However, if the same set of gradually increasing glutamatergic inputs arrive onto a dendritic membrane equipped with K^+^ channels (Fig. 3C–iii), then the rate of the EPSP amplitude increase would be markedly compressed (Fig. 3C–iv). K^+^ channels begin to activate at a certain voltage threshold, depicted by a blue horizontal line (Fig. 3C–iv, **V**_**act**_). Any further increase in dendritic voltage above **V**_**act**_ is strongly suppressed by a bold increase in the K^+^ current amplitude. In addition to the *amplitude compression* (shallow input-output (I–O) function), activation of these K^+^ channels also causes a rapid termination of the EPSP voltage waveform (Fig. 3C–iv, inset, compare red and blue trace). Interestingly, K^+^ current curtails EPSP amplification near threshold in inhibitory interneurons but not in pyramidal cells (Fricker and Miles, 2000). In summary, three biophysical properties of PV+ cells ([i] reverse Rm gradient (Norenberg et al., 2010); [ii] functional high-threshold rapid-activation K^+^ channels (Fricker and Miles, 2000; Hu et al., 2010); [iii] shallow (low-gain) I–O function (Kriener et al., 2022)), combined together provide strong basis for the linear dendritic integration, discussed in section 3.7.

*Nonlinear dendritic properties in PV+ interneurons*. Using exogenous glutamate, researchers attempted to trigger classical NMDA-dependent spikes (Schiller et al., 2000) in dendrites of PV+ interneurons (Chiovini et al., 2014; Cornford et al., 2019). The results of these studies indicated that some forms of nonlinear Ca^2+^ signaling can be achieved with gradually increasing stimulation, attributed to activation of L-type Ca^2+^ channels, Ca-permeable AMPA receptors, and Ca-induced Ca-release (Camire et al., 2018; Chiovini et al., 2014). However, compared to the PC spiny dendrites, which trigger robust glutamate-mediated local spikes (Antic et al., 2010; Dembrow and Spain, 2022), the PV+ interneuron dendrites exhibit very weak forms of NMDA-dependent nonlinearities (Cornford et al., 2019). Furthermore, a very large fraction of PV+ interneuron dendrites, in the same cell, show clear linear or even sublinear local responses (Cornford et al., 2019; Tzilivaki et al., 2019). In summary, synaptic integration in the PV+ interneuron dendrites is predominantly linear (Kriener et al., 2022).

### 3.7 Linear dendritic integration of afferent inputs

It has been proposed that: [i] spike synchrony, [ii] excitatory synaptic input power at gamma frequencies, and [iii] excitatory synaptic input correlation between neighboring cells, are much lower in excitatory PCs than in the inhibitory PV+ interneurons of the same brain region, because of the cell-type specific dendritic filtering of excitatory inputs (Salkoff et al., 2015). Dual site recordings and current injection protocols in dendrite and cell body of PV+ interneurons, showed that the gain of the I–O relationship for *dendritic-drive* was notably lower than for *somatic-drive* (Kriener et al., 2022). A shallow slope of the dendritic I–O relationship (Fig. 3C–iv) assured that large changes in current input produced only minimal changes in the cell body spiking. This means, a PV+ cell is less sensitive to input fluctuations if input is received on dendrite compared to an input received on the soma.

Computational network models of PV+ interneurons tested a similar scenario but instead of current injection, they used EPSPs. The computer model showed that synaptic input arriving on the dendrites strengthen the robustness of gamma synchrony in heterogeneous networks, and this conclusion held for a wide range of neuronal and network parameters (Kriener et al., 2022). Several biophysical properties may explain this interesting finding. **First,** EPSP attenuation along the dendrites is very strong, producing tiny membrane-potential fluctuations in the cell body, leading to more regular spiking despite large fluctuations in the afferent input drive. **Second,** PV+ distal dendritic segments strongly depolarize when excited by synaptic inputs (Norenberg et al., 2010). Strong local dendritic depolarizations could bring the dendritic membrane closer to the EPSP equilibrium potentials (in the case of glutamatergic AMPA and NMDA receptors, the EPSP equilibrium potential is around −10 mV). Once the dendritic membrane potential approached the EPSP equilibrium potential, further increases in afferent synaptic drive are very ineffective, because the local driving force has been depleted ((Tran-Van-Minh et al., 2015) their figure 2A) – see also *dendritic saturation* in (Bush and Sejnowski, 1994). Large EPSPs in distal dendrites reduce the I–O gain, because they prevent additional EPSPs to influence the PV+ cell output. Some classes of excitatory inputs (e.g. thalamocortical) release multiple vesicles at one synaptic contact (Bagnall et al., 2011), which causes sublinear summation (Hu et al., 2010; Tamas et al., 2002), due to the reduction in driving force (Bush and Sejnowski, 1994). **Third**, dendritic spikes cause dramatic changes in the neuronal output of PCs (Larkum et al., 1999; Milojkovic et al., 2004), while PV+ cell dendrites lack strong regenerative events such as Na^+^ and large-amplitude Ca^2+^ spikes (Cornford et al., 2019; Hu et al., 2010) - but see (Camire et al., 2018; Chiovini et al., 2014). It is likely that dendritic Ca^2+^ transients mediated by ionic fluxes through AMPA receptors and internal calcium release (Camire et al., 2018; Francavilla et al., 2019) have relatively small impact on electrical signaling in the PV+ cell body/axon. This apparent lack of proper (PC-like) dendritic electrogenesis in PV + interneurons [regenerative voltage potentials are weak in large number of PV+ dendritic branches (Chiovini et al., 2014; Camire et al., 2018; Cornford et al., 2019; Tzilivaki et al., 2019)] eliminates a powerful source of output-fluctuations, which normally occur in cortical PCs when strong dendritic electrogenesis is successfully triggered (Antic et al., 2010; Larkum et al., 1999; Milojkovic et al., 2004; Schiller et al., 2000). **Fourth**, in dendrites lacking voltage-gated Na^+^ and Ca^2+^ channels, high-threshold, fast-activating K^+^ conductances dominate the landscape (Cornford et al., 2019; Hu et al., 2010; Rudy and McBain, 2001). It has been argued that, in addition to a rapid dendritic saturation (loss of driving force), dendritic K^+^ currents also act to decrease the I–O gain, by actively opposing dendritic depolarization (Hu et al., 2010). K^+^ channel activation makes PV+ cells less sensitive to clustered excitatory input, because clustered excitatory input produces large depolarizations which activate K^+^ channels efficiently. On the other hand, the PV+ cell's sensitivity to distributed input is not impaired by K^+^ channels, because small-amplitude (distributed) excitatory inputs minimally activate these high-threshold K^+^ channels (Hu et al., 2010). In computational models of PV+ interneurons, dendritic K^+^ channels dampen input heterogeneities, and thereby enhance spike synchrony (Kriener et al., 2022).

In summary, the dendritic tree of PV+ interneurons is built to reduce impact of the fluctuations in afferent drive, which in turn promotes interneuron network synchrony (Kriener et al., 2022). PV+ cell dendrites not only buffer spatial input heterogeneities but also temporal heterogeneities in a variety of model network architectures, leading to high neuronal synchrony. PV+ cell dendrites scale down the gain of the I–O relationship and reduce the cell's sensitivity to afferent input fluctuations. These new experimental and modeling data suggest that care should be taken when simplifying inhibitory neurons as point neurons without dendrites, in network models.

### 3.8 Short membrane time constant (tau)

For experimental estimates of tau, typically, neurons are depolarized (or hyperpolarized) by rectangular current pulse (Fig. 3D–i, curr. inj.) and the resulting membrane depolarization (charging curve) is fitted with an exponential function (Fig. 3D–i, dashed black line) to arrive at membrane time constant (tau) values interpreted as “*apparent tau in the presence of voltage-dependent conductances*”, as discussed by (Koch et al., 1996). PV+ FS interneurons possess relatively short tau in the order of <5 ms (Galarreta and Hestrin, 2002; Hasenstaub et al., 2005; McCormick et al., 1985) reviewed in (Hu et al., 2014).

As a fundamental passive neuronal property, tau affects the rise and decay times of voltage transients (Koch et al., 1996). Excitatory postsynaptic potential (EPSP) is one type of a neuronal voltage transient strongly affected by tau. Short tau (in combination with strong dendritic K^+^ current) promotes faster EPSPs waveforms. EPSPs with shorter waveforms render de-synchronized dendritic afferent activity completely ineffective (Fig. 4A), while they reward highly synchronized inputs with generation of axonal AP (Fig. 4B).

EPSPs with faster rise times are more powerful activators of voltage-gated Na^+^ currents due to the Na^+^ channel activation kinetics being faster than the inactivation kinetics (Azouz and Gray, 2000; Hodgkin and Huxley, 1990; Wickens and Wilson, 1998). If a rising slope of an EPSP were to be very slow, during the rising slope a significant number of Na^+^ channels would enter an inactivated state before the AP threshold is reached, and the neuron(s) may not fire despite significant depolarization (depolarization block). To achieve robust AP initiation, a near-threshold voltage transient (e.g., EPSP) needs to rise steeply. Shortening of the membrane tau facilitates EPSP-induced Na^+^ channel activation, thus reducing time-delay to threshold (Fig. 3D–ii, compare orange vs. turquoise trace). In summary, short tau is an important PV+ cell adaptation allowing faster voltage responses and faster electrical rhythms. A PV+ interneuron tau (less than 5 ms) in comparison to pyramidal neuron tau (∼15 ms), results in: [i] faster rise time of EPSP; [ii] faster initiation of APs (Fig. 3D–ii, orange trace); and [iii] less attenuation of higher frequencies in PV+ interneurons.

Each great simple rule such as “*short tau determines EPSP-to-AP time delay*” has a twist. In the CNS neurons receiving on proximal dendrites many EPSPs simultaneously, the time delay between onset of EPSP barrage and AP generation (dT) is notably shorter than the tau (Koch et al., 1996). Depending on the synapse clustering in space (segregation) and in time (synchronization), the threshold for spike generation can be reached in a fraction of tau. For example, in a neuron whose tau was found to be ∼20 ms, a strong and synchronous synaptic input can generate an AP only 3–4 ms after the onset of an EPSP barrage. On the other hand, if the EPSP input is temporally and/or spatially becoming more and more dispersed (in particular toward distal synapses), then dT will begin to be more and more influenced by the tau (Koch et al., 1996).

B. After Axon

In this section, we discuss properties of PV+ interneurons, deemed important for effective initiation and propagation of action potentials (APs). In PV+ interneurons, AP initiation and propagation are tuned to assure: [i] efficient transfer of fast synaptic rhythms into the PV+ interneuron output (GABA synapse), [ii] very short time delay between afferent synaptic input in dendrite and GABA release in axon terminal, as well as [iii] powerful inhibition of the postsynaptic targets.

### 3.9 High frequency AP firing - fast-spiking

In PV+ interneurons, active membrane properties involved in AP generation are well suited to spiking at higher frequencies – *fast*-*spiking* (Fellous et al., 2001). By combining intracellular staining, PV+ immunohistochemistry, or PV-Cre technology, researchers directly demonstrated that fast-spiking cells contain PV (Kawaguchi et al., 1987; Pfeffer et al., 2013). Neocortical PV+ interneurons can generate APs at a wide range of frequencies with little spike frequency adaptation (Connors and Gutnick, 1990; Kawaguchi and Kubota, 1997; Nowak et al., 2003). Many interneurons show fast-spiking (FS) phenotype. There is a spectrum of spiking speeds, with PV+ GABA-releasing basket cells representing the functional extreme (Bereshpolova et al., 2020; Resnik and Polley, 2017).

To spike at high frequency and fit many APs into a small unit of time, a neuron needs APs as brief as possible. In PV+ cells, duration of an individual AP is very short (∼0.4 ms). In fact, their AP is the briefest among neurons of cerebral cortex (McCormick et al., 1985; Mountcastle et al., 1969). Brief AP suggests rapid deactivation of the voltage-gated Na^+^ (inward) current and simultaneous rapid activation of the voltage-gated K^+^ (outward) current. These two rapid currents are built into PV+ interneurons at high K-to-Na ratio. The dominance of the K^+^ conductance over the Na^+^ conductance is deemed necessary for fast-spiking (discussed in (Hu et al., 2014)). To achieve high K-to-Na ratio, the PV+ cell has to pack a lot of K^+^ channels into the plasma membrane. Indeed, glass electrode measurements performed on the same recording setup, same capillary glass, and same conditions, show that K^+^ currents of inhibitory cells are larger than those of PCs (Fricker et al., 1999; Martina et al., 1998). Antibody staining and single-cell PCR revealed that both Kv3 (Kv3.1, Kv3.2) and Kv4 (Kv4.2, Kv4.3) subunits are abundant in interneurons (Chow et al., 1999; Martina et al., 1998). The gating properties of Kv3 channels, in particular: [i] high activation threshold, [ii] fast activation, [iii] fast deactivation, and [iv] lack of inactivation; are essential for fast-spiking (Hu et al., 2014; Rudy and McBain, 2001). A premature Kv3 channel closure may potentially lead to incomplete membrane repolarization, thus preventing sustainable fast-spiking. To solve this, Kv3.1 b channels produce resurgent K^+^ current which acts to terminate individual APs. This resurgent current in the Kv3 channel class, results from a unique combination of steep voltage-dependent gating kinetics and ultra-fast voltage-sensor relaxation (Labro et al., 2015). In summary, a high density of Kv3.1 channels contributes to: [i] short-duration action potentials, [ii] fast afterhyperpolarization (AHP), [iii] brief refractory periods, and [iv] high K-to-Na conductance ratio; thus enhancing the capability in these neurons for high-frequency firing (Lien and Jonas, 2003).

Suppressing the outward K^+^ current increases the variability in latency of synaptically-induced firing in interneurons (Fricker and Miles, 2000). Selective block of fast delayed rectifier K^+^ channels, presumably assembled from Kv3 subunits, by 4-aminopyridine (4-AP), reduced substantially the AP frequency in interneurons (Martina et al., 1998). Low tetraethylammonium (TEA) concentrations (∼1 mM), which block only Kv3.1-Kv3.2 channels, strongly impaired afterhyperpolarization and high-frequency firing (Erisir et al., 1999) and caused broadening of APs (Lien and Jonas, 2003). Subtraction of Kv3 conductance by *dynamic clamp* mimicked the effects of the pharmacological Kv3 channel blockers (Lien and Jonas, 2003).

K^+^Channel Knockout Experiments. PV+ interneurons in deep cortical layers of a Kv3.2 knockout mouse have broader spikes and sustain significantly lower firing frequencies. Such differences are not observed in PV+ interneurons in superficial layers L2 – L4 of Kv3.2 knockout mouse, where Kv3.2 proteins are only weakly expressed. These data indicate that Kv3.2 channels regulate AP half-width and fast-spiking in infragranular PV+ interneurons (Lau et al., 2000). In homozygous Kv3.1 (−/−) mice, there is a dramatic, several fold, increase in both absolute and relative spectral power of the gamma frequency oscillations (Joho et al., 1999). This result is unexpected and it is contrary to two ideas: [1] the idea that Kv3.1 is essential for fast-spiking in PV+ interneurons, and [2] the idea that fast-spiking in PV+ interneurons is essential for fast cortical oscillations (Rudy and McBain, 2001).

Afterhyperpolarization (AHP). In PV+ interneurons, large amplitude AHPs follow APs (Kawaguchi, 1993b; Koyanagi et al., 2014; McCormick et al., 1985). The kinetics of the PV+ AHP appear to be optimal for recovery of Na ^+^ channels from inactivation and minimal refractory period (Kawaguchi, 1993b). A shorter AHP than those recorded in PV+ cells would not permit sufficient recovery of Na ^+^ channels from inactivation, leading to longer AP refractory periods (discussed in Jonas et al. (2004)).

Refractory Period. Action potential refractory period in PV+ neurons (∼30 ms) is significantly shorter than that in PCs (∼90 ms) (Hasenstaub et al., 2005). Such a short refractory period allows PV+ interneurons to outpace the PCs, as well as to insert spike doublets (Kawaguchi and Kubota, 1997). Spike doublets in PV+ interneurons are considered important for global synchronization (Traub et al., 1996).

Slow inactivation constant of Na^+^channels. Two important features of the voltage-gated Na current are: [1] the speed of inactivation (inactivation time constant) and [2] the speed of recovery from inactivation. Either alone, or in combination, these two features affect the AP half-width, refractory period, spike frequency adaptation, and spike threshold accommodation. To achieve narrow (brief) spikes (as in PV+ cells), one needs fast Na^+^ channel inactivation (faster termination of AP). However, in PV+ cells, voltage-gated Na^+^ channels have a relatively slow inactivation constant (19 ms at −55 mV), whereas this time constant is two-time faster (9 ms) in the neighboring PCs (Martina and Jonas, 1997). Slow inactivation constant! - this is counterintuitive! A full cycle ([i] activation → [ii] inactivation → [ii] escape from inactivation) should be completed in a short time to achieve fast-spiking. Martina and Jonas (1997) provide an explanation: To achieve fast-spiking (as in PV+ cells), the Na^+^ channel inactivation dynamics should be slow (to preserve the pool of activatable Na^+^ channels), while the recovery from Na^+^ channel inactivated-state back to ready-to-be activated state, needs to be fast (to increase the pool of activatable Na^+^ channels). Indeed, under the same experimental conditions, the time course of recovery of Na^+^ channels from inactivation is faster in PV+ cells compared to PCs (Martina and Jonas, 1997). Slower *inactivation* and faster *recovery* likely enable continual Na ^+^ channel availability during spike trains and prevent spike frequency adaptation and spike threshold accommodation (Martina and Jonas, 1997; Tremblay et al., 2016).

Type 2 firing. In fast-spiking PV+ inhibitory interneurons, regular firing is supported over a wide range of frequencies, yet there is a clear discontinuity in their evoked spike frequency vs current injection (F–I) relationship at some current threshold. This type of F–I is called *discontinuous* or *Type 2*, to differentiate from *Type-1* found in pyramidal cells. The *Type-1* F–I relation is continuous at threshold (Tateno et al., 2004). FS neurons are unable to support sustained periodic firing below a critical frequency, e.g. in the range of 10–30 Hz. Very close to threshold, PV+ interneurons switch irregularly between bursts of periodic firing and subthreshold oscillations (Tateno et al., 2004). The dynamics of PV+ interneuron spiking appears to be well suited to detecting, following, and maintaining higher-frequency inputs, most notably the gamma frequency inputs (greater than 30 Hz) (Tateno et al., 2004; Tikidji-Hamburyan et al., 2015).

In summary, [i] shorter membrane time constant of PV+ interneurons, [ii] features of the Na^+^ channel function (rapid activation, slow inactivation, and rapid escape from inactivation); [iii] features of the K^+^ channel function (high activation threshold, fast activation, fast deactivation, lack of K^+^ channels inactivation, and high K/Na conductance ratio), [iv] brief AP (a record holder in the cortex), [v] short refractory period (a record holder in the cortex), [vi] periodic burst firing; when combined allow PV+ interneurons to outpace other cells in the cortex and force their own spikes into the early phase of the emerging, or ongoing, cortical rhythm.

### 3.10 Intrinsic membrane resonance in the gamma-band

There are three suggested mechanisms for the 40 Hz preference in PV+ interneurons, but these mechanisms are not sharply delineated - they may merge, blend, and cooperate (Moca et al., 2014). Besides this important point (several mechanisms may blend and overlap significantly to the point where it is not clear which mechanism is in charge), another important point is that whichever mechanism one adopts as the primary mechanism of gamma band oscillation, it is unavoidably going to be based on the interplay between 5 players: [i] chemical synapses; [ii] electrical synapses; [iii] wiring between neurons (connectome); [iv] which channel-species comprise the set of intrinsic membrane conductances, and [v] conductance densities.

We begin with intrinsic membrane properties which force neurons to have preferences for specific frequency ranges in electrical signaling - resonance (Dembrow et al., 2010; Hutcheon and Yarom, 2000; Kelley et al., 2023). An isolated PV+ interneuron (e.g. in a silent brain slice) is inclined to fire at 40 Hz (Fig. 3E–i). The response of a PV+ cell membrane to the injection of broad band fluctuations shows less decrement of higher frequencies; around 40 Hz (Fellous et al., 2001). Outside the neocortex, fast-spiking interneurons also show an inherent tendency to resonate around 40 Hz (Bracci et al., 2003; Pike et al., 2000; Sciamanna and Wilson, 2011). Intracellular injections of colored noise (Hasenstaub et al., 2005) revealed that in many neocortical PV+ interneurons, the transfer of frequencies peaked near the gamma point (Fig. 3E–i, inset, white rectangle). Here, we dubbed this mechanism as interneuronal gamma, IG (Fig. 3E–i) to point out the central role of the interneuronal (IN) membrane, but it may also be the case that a more appropriate name for this mechanism would be RING (Resonance INduced Gamma). The RING Theory was developed from a need to explain findings that the oscillation frequency is stable and bound within a relatively narrow range, even when in computational models, the network elements are subjected to various modulations of their electrophysiological properties (membrane time constant, synaptic time constant, synaptic strengths, and magnitudes of incoming inputs). It was suggested that the stability of the fast network oscillation relies on the resonant properties of individual PV+ interneurons. Computational studies found that resonant interneurons enhance the ability of oscillating networks to maintain a uniform frequency in the presence of variable levels of extrinsic drive (Baroni et al., 2014; Moca et al., 2014). In summary, PV+ interneuron membrane resonance (Fig. 3E–i) enhances stability, robustness, and power of network gamma oscillations.

Besides the resonant intrinsic membrane properties of the PV+ interneurons, an induction of electrical gamma oscillation may also be based on the neural network dynamics, connectome, and synaptic properties. Several groups suggested that fast-spiking PV+ interneurons generate a specific cortical electrical rhythm at ∼40 Hz (gamma frequency band), either through interaction with other PV + cells (*The ING Theory*, Fig. 3E–ii), or through interaction with excitatory pyramidal cells (*The PING Theory*, Fig. 3E–iii). *The PING Theory* is popular because excitatory PCs lead FS PV+ interneurons by ∼5 ms, on average (Hasenstaub et al., 2005; Moca et al., 2014). The time required to complete one full cycle, from PC discharge to fast-spiking PV+ interneuron discharge, to inhibition of postsynaptic neurons (both pyramids and interneurons), occurs within approximately 25 ms, thereby promoting a preferred frequency range in the cortex (and hippocampus and amygdala) of 40 ± 10 Hz (Salkoff et al., 2015). The arguments for several network theories (Fig. 3E) are numerous (Borgers et al., 2005; Cardin et al., 2009; Salkoff et al., 2015; Sohal et al., 2009; Ter Wal and Tiesinga, 2021; Traub et al., 1996, 2000), and will not be resolved in this review.

Regardless of the precise cell-type interactions (Fig. 3E–ii vs Fig. 3E–iii), temporally structured synaptic inputs into PV+ interneurons can be enhanced by subthreshold oscillations and resonance phenomena in the gamma range (Fig. 4D, 40 Hz) (Fellous et al., 2001; Hasenstaub et al., 2005; Moca et al., 2014; Whittington and Traub, 2003). One important observation is that inhibitory GABAergic interneuron networks seem to generate rhythmic synchronization regardless of whether their excitatory drive is rhythmic. The gamma oscillations evoked by PV+ interneuron activity are an emergent property of the circuit (resonant circuit property) and do not require exclusive drive in the gamma range (Cardin et al., 2009; Neymotin et al., 2011).

Optogenetic silencing of PV+ interneurons show that the integrity of the PV+ circuit is critical for the gamma oscillations (Antonoudiou et al., 2020; Sohal et al., 2009). However, one study suggested that emergent gamma oscillations occur robustly in multiple cell types and may thus be a generic feature of all inhibitory networks throughout the brain (Sohal and Huguenard, 2005).

Extracellularly recorded local field potential (LFP) are predominantly comprised of the electrical fields from PC dipoles, but PCs follow the synchronized activity of interneurons, hence LFP recordings can indirectly reveal the actions of interneurons (Buzsaki and Wang, 2012). Rhythmic synchronization of the interneurons imposes synchronized rhythmic inhibition onto the local PCs, so that the end result is: robust rhythmic activity of the entire PC network (Fries et al., 2007). Pyramidal neurons typically discharge a few milliseconds before the phase of the LFP. Dual intracellular recordings show that pyramidal excitatory cells fire their spikes ∼5 ms before PV+ interneurons fire their spikes (Hasenstaub et al., 2005; Moca et al., 2014). Intracellular recordings in PCs show sequences of EPSP–IPSP (Isaacson and Scanziani, 2011; Salkoff et al., 2015). These findings fit the PING theory – a theory in which an excitation from PCs onto the PV+ cells is the main engine of the oscillation (Fig. 3E–iii).

### 3.11 Myelinated axon – only PV+ interneurons have it

Among all cortical inhibitory GABAergic interneurons, only the PV+ subclass possesses a myelinated axon (Stedehouder et al., 2017). To date, myelinated axons have exclusively been observed in PV neurons. While other types of isocortical interneurons (SST, VIP) may potentially possess myelinated axons, they have not yet been detected. In neocortical gray matter, myelin is thought to mostly wrap excitatory axons of projection neurons (PCs). However, about 50% of all myelinated axons in layers L2/3, and around 25% of myelinated axons in L4 originate from local PV+ basket cells. This is an interesting finding. Why do short-axon local interneurons (PV+ cells) need myelin?

The axon of a PV+ interneuron becomes myelinated soon after exiting the cell body (usually within 20–50 μm) (Micheva et al., 2016). PV+ interneuron myelination most commonly involves the proximal axon (Somogyi et al., 1982), it consists of internodes interspersed by axonal branch points, and occurs predominantly on longer interbranch segments (Stedehouder et al., 2017). It was suggested that oligodendrocytes (the cell type which produces myelin in the brain) can recognize the class-identity of individual types of cortical interneurons that they target. Some oligodendrocytes disproportionately myelinate the axons of inhibitory interneurons, whereas other oligodendrocytes primarily target excitatory axons belonging to PCs (Zonouzi et al., 2019). Both, the internodes (the myelinated portion between two nodes of Ranvier) and the actual nodes of Ranvier (naked portion with high density of channels) are shorter in GABA-releasing axons compared to non-GABA axons regardless of a cortical layer examined (Micheva et al., 2016).

Myelin deficit affects firing frequency. In theory, a myelinated axon can shorten the time delay between the cell body spike on one side of the axon, and synaptic release at the PV+ terminal, on the other side of the axon. But PV+ axons are confined to a limited space (Gouwens et al., 2020; Kawaguchi, 1993a; Kawaguchi and Kubota, 1997), so there is no pressure to speed up AP propagation with sheaths of myelin. Only 4% of the total axon length of PV+ interneurons is myelinated, yet, myelin loss, or myelin dysfunction, can cause dramatic changes in: [i] PV+ firing frequency, [ii] inhibitory circuit function, and [iii] behavior. Myelination of PV axons is required to consolidate fast inhibition of pyramidal neurons and enable behavioral state-dependent modulation of local circuit synchronization (Dubey et al., 2022). Myelination defects of PV+ interneurons cause a significant reduction in the AP firing frequency, but other properties such as PV+ interneuron input resistance, resting potential, afterhyperpolarization, and AP peak amplitudes, are not affected by myelination defects (Benamer et al., 2020).

Myelin deficit affects synaptic function. Axonal myelination appears to influence synaptic functions (Dubey et al., 2022). For example, inactivation of the synapse between PV+ cells and oligodendrocyte precursor cells (OPCs) during early postnatal development, resulted in myelination defects of PV+ cells that were associated with a strong reduction of PV+ synaptic connections with spiny stellate cells (inhibitory synapse) causing a significant reduction in the feedforward inhibition at this major sensory input level (L4) (Benamer et al., 2020). In this scenario, GABAergic interneurons and oligodendroglia are reciprocal partners during development (Orduz et al., 2019). Beyond electrical conduction, myelin around the PV+ axons can regulate the: [i] morphology of proximal axon; [ii] high frequency of AP discharges; and [iii] connectivity of PV+ interneurons with local excitatory neurons (Benamer et al., 2020; Zonouzi et al., 2019). An impaired feedforward inhibition at the cortical circuit level is manifested by an impaired whisker-dependent texture discrimination behavior (Benamer et al., 2020).

Axonal adaptations. In PV+ cells, small diameter and extensive branching of the axon should limit AP generation and propagation. To compensate for a small axon diameter and extensive axon branching, PV+ cells developed several adaptations. **First**, the density of Na^+^ channels is high (*supercritical*) in PV+ axons. “Supercritical” means more than necessary for generation of one AP (Hu and Jonas, 2014). Confocally-targeted subcellular patch-clamp measurements revealed a stepwise increase of Na^+^ conductance density from the soma to the proximal axon. This already very high density of Na^+^ channels in the proximal axon, is further increased in the more distal sections of the same axon (Hu and Jonas, 2014). **Second**, the distance between the axon hillock and the AP initiation site (AP trigger zone, TZ), as well as the actual length of the TZ, are significantly shorter in PV+ interneurons (Hu and Jonas, 2014) compared to pyramidal cells (Popovic et al., 2011). In other words, the AP TZ of PV+ interneuron is very close to the cell body. It has been shown that axonal TZ is not fixed at one precise distance from the cell body, but instead it may slide along the axon (as part of a homeostatic mechanism) to adjust the excitability of the cell. The closer it approaches the cell body the more excitable the neuron becomes (Kuba et al., 2006). **Third**, a very high density of Kv3 channels. The impacts of Kv3 channels on AP duration, initiation, refractory period, and spiking frequency are discussed in section 3.9. **Forth**, myelin wrap is developed to concentrate (to corral) voltage-gated channels in precise location (node of Ranvier) (Duflocq et al., 2011), reduce membrane capacitance (cm) and increase membrane electrical resistance (rm) of axonal segments under the myelin sheath. **Fifth**, myelination ensues in the proximal part of the axon where fast APs need to be initiated at high frequency. Fast-spiking is supported by myelin and myelin-mediated concentration of voltage-gated channels in the axon (Duflocq et al., 2011). The transfer of the AP pattern from the AP TZ to the GABA-releasing synaptic terminals on multiple axonal branches is assured by a smaller diameter of the daughter axon branch compared to the parent axon branch, in combination with gradually increasing Na^+^ channel density in the distal axon. In summary, the overtly high Na^+^ channel density compensates for unfavorable morphological properties of PV+ cell axons (small diameter, extensive branching, and high bouton density). Supercritical Na^+^ channel density increases the conduction velocity, resulting in shorter time delays between AP initiation and onset of inhibitory postsynaptic potential (IPSP) arriving on PCs (Hu and Jonas, 2014; Tremblay et al., 2016). **Sixth**, PV+ cells fire very often (on any kind of change in the sensory input) (Sohya et al., 2007), and when they fire, they fire with high frequency, thus generating higher metabolic demands compared to pyramidal cells. Pyramidal cells fire only if the object class matches their job description and when they do fire, they typically fire at a low frequency. One potential role of myelin at PV+ cell proximal axon is reduction in metabolic needs. Myelin improves the energy efficiency of AP propagation (Hartline and Colman, 2007). Also, myelin provides metabolic support to the axon by supplying energy molecules (Lee et al., 2012; Micheva et al., 2016). In summary, myelination of the PV+ axon may be a critical feature for the healthy excitation-inhibition balance (Bhatia et al., 2019), as well as for the proper information processing in the brain (Fig. 1B).

PV+ IN myelination is conserved in humans. Using acutely resected cortex from human patients, Stedehouder et al. (2017) showed that all of the human PV+ interneurons examined (8/8 cells), but none of the non-fast-spiking cells (0/3 cells), exhibited myelinated axons. Similar to their finding in mice, PV+ interneuron myelination in the human neocortex was biased towards proximal axonal segments (Stedehouder et al., 2017). Interestingly, mouse and human neocortical interneurons follow similar morphological rules guiding the topography of axonal myelination. The topography of myelination along individual PV+ axons is strongly predicted by the joint combination of axonal diameter and interbranch distance (Stedehouder et al., 2019). The conserved feature of both the mouse and human brain is likely a consequence of the evolutionary pressure to conserve an efficient mechanism to ensure that a high proportion of action potentials pass through myelinated segments, thereby potentially enhancing conduction velocity, fidelity, and metabolic support.

### 3.12 Small synaptic delay

Time delays affect generation, transmission, and processing of information between interconnected neurons in the cerebral cortex. AP propagation through the axonal tree is influenced by conduction velocity, which is sensitive to axon diameter, axon branch points, presence or absence of myelin, composition, and density of voltage-gated conductances. Changes in the AP propagation delay(s) can affect communication between connected neurons by modulating both the temporal and spatial properties of both: [i] the presynaptic- and [ii] postsynaptic activity patterns.

*Axonal propagation delays* are deemed necessary for generation of nearly synchronous responses in postsynaptic neurons through axons with multiple collaterals and hence multiple postsynaptic target neurons. For example, the same cortical projection neuron (e.g. L5 pyramidal cell) sends axons to proximal and remote brain structures, where the conduction velocities of these axonal pathways can vary up to threefold (Chomiak et al., 2008). Computational studies suggest that propagation delays modify weight and neuronal dynamics by shaping the emergent functional and structural properties of plastic neuronal networks (Madadi Asl et al., 2018).

Networks of reciprocally connected inhibitory neurons are often involved in the generation of fast brain rhythms, where rhythms arise through the synchronization of neurons and their entrainment in a regular firing pattern. PV+ axon conduction delays can stabilize neural network synchrony in several ways, including: [i] neutralization of discontinuity introduced by strong inhibition; [ii] favoring synchrony in the case of noisy bistability; and [iii] avoiding an initial destabilizing region of a phase resetting curve (Wang et al., 2012).

Varying Synaptic Delay in Model. An inhibitory network model developed for reproducing 160 Hz oscillations recorded in the cerebellar cortex of transgenic mice deficient for calretinin and calbindin, produced some interesting conclusions that may potentially apply to cortical PV+ interneurons. Resonant synchronization can occur in computer-simulated networks of cerebellar inhibitory neurons if the synaptic current has a delayed onset, reflecting finite spike propagation and synaptic transmission times (synaptic delays). By varying the axonal (synaptic) delay of the inhibitory connections, networks with a realistic synaptic kinetics can be tuned to frequencies from 40 Hz to 200 Hz. These results suggest that axonal (synaptic) delay is one of the primary parameters controlling the oscillation frequency of the cerebellar inhibitory networks (Maex and De Schutter, 2003). It is possible that similar computational studies centered on the cortical cytoarchitectonic may arrive at two conclusions: [1] *Synaptic delay* in the PV+ interneuron connections is a cardinal parameter controlling fast cortical oscillations; and [2] *Tunable synaptic delays* should be included into the list of very important factors for generation of fast cortical oscillations (Buzsaki and Wang, 2012; Ter Wal and Tiesinga, 2021; Via et al., 2022).

### 3.13 Inhibitory autapses – autoinhibition – rebound spiking

Gamma-band rhythmogenesis is inextricably tied to perisomatic inhibition (Buzsaki and Wang, 2012). PV+ cells inhibit each other with: [i] larger conductances, [ii] smaller decay time constants, and [iii] shorter synaptic delays (Fernandez et al., 2022). One powerful form of inhibition occurs when the axon of a PV+ interneuron makes a collateral branch which swings back, returns to the cell body of the same neuron from which it originated, and delivers a GABAergic synapse – a self-synapse (Fig. 4E, Auto-synapse). Perisomatic autaptic inhibition is common in both human and mouse PV+ interneurons of the supragranular neocortex, where they efficiently control discharge of the PV+ interneurons (Szegedi et al., 2020; Tamas et al., 1997a). Autaptic transmission represents the most powerful inhibitory input of PV+ cells in neocortical layer 5 (Deleuze et al., 2019).

Postinhibitory Rebound.*Dynamic clamp* experiments routinely achieve autapse-induced firing in cortical interneurons when a computer detection of an AP (determined by a crossing of the −20 mV threshold) is followed by an artificial (computer-generated) inhibitory postsynaptic conductance with a 2 ms delay (Tikidji-Hamburyan et al., 2015). How can an inhibitory input induce initiation of an AP in the postsynaptic cell? Apparently, inhibitory input hyperpolarizes the recipient (postsynaptic) cell. Following cessation of the inhibitory conductance, the membrane potential surges upward, from hyperpolarized potential toward the resting membrane potential. If this depolarizing trajectory is of sufficient amplitude and steepness, Na^+^ channels activate at sufficient numbers and trigger an AP (rebound firing), in many neuron types (Bevan et al., 2000; Buhl et al., 1994; Deuchars and Thomson, 1995). Rebound is a paradoxical excitation in which, following a period of strong hyperpolarization below the resting membrane potential, the membrane potential briefly rebounds to a more depolarized level resulting in firing spikes. Rebound spiking is thus triggered by inhibition and not by excitatory inputs.

In summary, PV+ autapses bring two important features to PV+ interneurons. First, the initial outburst of APs is quickly shut down. Second, the same force which terminates the old AP outburst, stimulates the next AP outburst. PV+ interneurons are designed to experience rapid alternating waves of inhibition, hence their opportunity for spiking lies in between two peaks of inhibition.

All cellular mechanisms for explaining interneuronal network gamma oscillations rely on alternating waves of inhibition and windows of opportunity for spiking. Allegedly, the *coupled oscillator models* (Wang and Buzsaki, 1996) are not sufficiently robust to heterogeneity in excitatory drive. Implementations of so-called *resonator neural models*, which exhibit: [i] Type-2 excitability (abrupt onset of firing at a threshold frequency (Tateno et al., 2004)) and [ii] postinhibitory rebound (rebound from powerful autapses), results in much greater robustness to heterogeneity that increases as the average participation in spikes per cycle, thus approximating the physiological levels (Tikidji-Hamburyan et al., 2015).

### 3.14 GABA release onto pyramidal cell (PC) body and axon

Perisomatic inhibition is critical for gamma oscillations (Buzsaki and Wang, 2012). Synchronization of cortical pyramidal neurons is substantially more effective by perisomatic inhibitory postsynaptic potentials (IPSPs) than dendritic excitatory EPSPs (Lytton and Sejnowski, 1991). Accordingly, PV+ interneurons show denser axonal arborizations near the somata of PCs (Kawaguchi and Kubota, 1997). PV+ chandelier cells innervate the axon initial segment of neighboring PCs, while PV+ basket cells target the soma and adjoining basal dendrites of PCs (Gouwens et al., 2020; Kawaguchi and Kubota, 1998; Tamas et al., 1997b; Thomson et al., 1996). Precise & strategic positioning of the PV+ synapses on- and near the axon initial segments of PCs, is designed to veto AP initiations in PCs (Somogyi et al., 1982).

To change membrane potential of the axon initial segment, or to shunt depolarizing currents away from the axon initial segment, inhibitory synaptic inputs must dock nearby, preferably on the axon initial segment itself. The prime example is a PV+ chandelier cell and the cartridge synapse it makes along an axon initial segment (Howard et al., 2005; Tai et al., 2014). Based on the size of the ammunition (big cartridge), and the precise subcellular targeting (directly on the AIS), one would expect that PV+ chandelier cell is the most effective veto-maker in the cortex. However, each great simple rule such as “*GABA blocks excitation*” has a twist. Instead of blocking AP initiation, a GABAergic input onto the axon may trigger AP initiation in some cases (Szabadics et al., 2006). Apparently, axons of some cortical PCs lack a certain type of an ion transporter, which causes unusually high concentration of Cl^-^ in the axon, and this pushes the local GABA reversal potential to more positive values. As a result, in some axons, responses evoked by *chandelier* synaptic contacts on the axon are depolarizing at resting membrane potential. In other axons, single spikes in presynaptic PV+ chandelier interneuron elicited postsynaptic action potentials in PCs with a >50% probability. This outcome was blocked by gabazine suggesting that it was exclusively mediated by GABA-A receptor currents. In the same study, under the same experimental conditions, GABA inputs from PV+ basket cells could not evoke APs in neighboring PCs, despite having a larger amplitude range than PV+ chandelier cells (Szabadics et al., 2006). In the primate cortex, the cartridges of chandelier-cell axons were most dense in layers L2, L3, and L4, but were not observed in the infragranular layers L5 and L6 (Williams et al., 1992), suggesting that chandelier cells do not “police” the projections to the colliculi, pons and medulla (L5), or projections to the thalamus (L6), but rather they aim to inhibit (or excite) cortico-cortical projections stemming from L2/3 PC axons.

Two interesting deductions arise from these findings: [i] A single chandelier cell contacts more than 100 PCs, therefore it is in a position to activate (excite) more than 100 PCs with a single spike of its own; and [ii] Chandelier cells do not target GABAergic interneurons; therefore, forward inhibition cannot oppose the intentions of the chandelier cells.

### 3.15 GABA release onto pyramidal cell (PC) dendrites

Despite popular belief that PV+ interneurons only impinge near cell bodies of cortical PCs, while non-PV interneurons inhibit dendrites, ample PV+ contacts are found in distal dendrites of PCs (DeFelipe et al., 2013). Similar to invertebrate neurons showing multiple spike initiation zones (Antic et al., 2000), cortical PCs also have multiple spike initiation zones in the same cell (Larkum et al., 2009). Therefore, to achieve effective vetoing of the PC activity, inhibition is required to be directed to both locations of regenerative spike generation (Pouille et al., 2013): [i] at the soma, where regenerative spikes are based on fast Na^+^ currents (Stuart et al., 1997), and also [ii] in the dendrites where regenerative spikes are based on currents with slower kinetic (Ca^2+^ and NMDA) (Antic et al., 2010; Larkum et al., 1999).

Can GABAergic synapses control dendritic spikes? Dendritic NMDA spikes are highly sensitive to dendritic inhibition (Doron et al., 2017; Rhodes, 2006). When impinging on its early phase, individual inhibitory synapses strongly, but transiently, dampen the NMDA spike; later inhibition prematurely terminates it. NMDA spikes in distal dendritic branches/spines are longer-lasting (Gao et al., 2021) and more resilient to inhibition, enhancing synaptic plasticity at these branches (Doron et al., 2017).

In summary, both perisomatic and dendritic inhibitory synapses are capable of impacting the I–O curve of cortical PCs. GABA-A activation onto the same dendritic compartment as the excitatory drive, produces a rightwards shift in the I–O function (subtractive gain control), versus GABA-A located proximal to the excitatory drive (near the cell body) which causes both a rightwards shift and also a reduction in the maximal firing rate (divisive gain control) (Pouille et al., 2013). Sparse weak inhibition can tune synaptic plasticity both locally at the dendritic branch level and globally at the level of the neuron's output (Doron et al., 2017).

### 3.16 Electrical synapses – gap junctions

In contrast to chemical synapses (e.g. glutamatergic, GABAergic), the electrical synapses (gap junctions) are void of synaptic delays and high metabolic demands, and as such they provide effective tools for synchronizing electrical activity among cells. CNS neurons couple to other CNS neurons at gap junctions formed by plaques of hemichannel pores composed of connexin proteins that allow ions to pass between neurons (Vaughn and Haas, 2022). Importantly, the current flows directly between neurons without relying on energetically costly neurotransmitters, or a proper (full-size) presynaptic spike – any membrane potential transient (negative or positive, subthreshold or suprathreshold) can contribute to this form of electrical communication. Based on Ohm's Law, net ionic current will flow through the gap junction channel proportional to the voltage difference between the presynaptic and postsynaptic neuron (Connors, 2017). Unlike chemical synapses, most electrical synapses conduct bidirectionally, allowing groups of neurons to rapidly share and distribute mutual excitations and inhibitions. This type of voltage sharing reduces neuron-to-neuron differences in either absolute voltage or neuronal electrical activity (spiking), hence it constitutes a very attractive mechanism for explaining synchrony between electrically coupled neurons (Connors, 2017; Vaughn and Haas, 2022).

Electrical synapse is a unique adaptation of cortical inhibitory interneurons. Electrical synapses strongly influence the electrical activity of adult cortical GABAergic interneurons (Ferrer and De Marco Garcia, 2022; Tremblay et al., 2016). This is in stark contrast to the adult cortical PCs, which comprise 80% of all cortical neurons, but rarely use electrical synapses for communication (but see (Draguhn et al., 1998; Schmitz et al., 2001)). The discovery of electrotonic coupling through gap junctions between cortical GABAergic neurons (Galarreta and Hestrin, 1999, 2002; Gibson et al., 1999) suggested early on that these cells may generate large, synchronous inhibitory potentials within cortical networks. Interestingly, electrical synapses (gap junctions) almost exclusively connect GABAergic neurons belonging to the same class (e.g. PV+) (Galarreta and Hestrin, 1999; Gibson et al., 1999; Landisman et al., 2002). Also, gap junction connections are more frequent between PV+ interneurons than between SST + interneurons (Fernandez et al., 2022). Parvalbumin (PV)-positive interneurons form dendritic gap junctions with one another, with the majority (∼85%) of dendritic gap junctions within 75 μm from the cell body (Shigematsu et al., 2019). The overwhelming majority of PV-to-PV electrical synapses are located on the cell body and near the cell body, on the most proximal segments of dendritic branches (Galarreta and Hestrin, 1999; Gibson et al., 1999; Tamas et al., 2000). Histological evidence for axonal gap junctions in the cortex is not as strong as for dendritic gap junctions, but electrophysiological studies and computer simulations have provided evidence for their existence in the neocortex (Traub et al., 2001). Interestingly, PV-PV axonal gap junctions operate in the amygdala (Muller et al., 2005).

Biphasic voltage change. Electrical synapses between PV+ interneurons are void of synaptic delays (current passes directly from pre-to post-synaptic element), which promotes PV+ interneuron network synchrony at high frequencies (Beierlein et al., 2000; Hormuzdi et al., 2001; Szabadics et al., 2001; Tamas et al., 2000). As electrical coupling is mostly mediated via perisomatic gap junctions (Tamas et al., 2000; Traub et al., 2003) (Fig. 4E), the time course of the gap junction potentials is fast, essentially following the time course of the presynaptic action potential (Fig. 4F, gap junc. potential). Two PV+ interneurons are coupled via both electrical (gap junction) and chemical (GABA) synapses, hence an action potential in the presynaptic PV+ interneuron triggers a biphasic sequence of excitatory (inward) – inhibitory (outward) currents in the postsynaptic PV+ cell (Fig. 4F, biphasic voltage change). Apparently, electrical coupling (gap junction) can selectively regulate the coherence of high-frequency network oscillations, whereas the time course of the chemical GABA_A_-receptor-mediated component can control both coherence and frequency (Bartos et al., 2002). Upon activation of presynaptic PV+ cells (Fig. 4E, purple IN), postsynaptic PV+ cells experience a sequence of depolarization and hyperpolarization; a biphasic voltage change (Fig. 4F) (Galarreta and Hestrin, 2001; Gibson et al., 1999). When a network of FS PV+ cells receives coherent excitatory inputs (Fig. 4B), the firing of PV+ cells will be promoted by signaling via electrical synapses (Fig. 4E, gap junction). If the same number of excitatory inputs arrive as a non-coherent wave (Fig. 4A, de-synchronized input), the firing of the PV+ cells will inhibit each other via their mutual GABAergic synapses (Fig. 4E, Synapse GABA). During that brief amount of time spent in a transient depolarization (Fig. 4F, gap junc. potential), before inhibitory hyperpolarization fully envelops (IPSP), PV+ cells can produce an AP, or even a doublet of APs (consequence of a super-short AP refractory period).

Syncytium of electrically coupled cells. PV+ GABAergic interneurons comprise 30–50% of all cortical GABAergic interneurons (Gonchar et al., 2007), giving them sufficient density to effectively propagate waves of electrical activity. An electrical signal generated in one PV+ interneuron propagates through a syncytium of PV+ interneurons, where individual cells are connected via abundant and reciprocal gap-junction coupling (Amitai et al., 2002; Hestrin and Galarreta, 2005). Electrical activity propagates between adjacent brain regions mostly through horizontal glutamate-mediated connections running through superficial cortical layers 1–2, or intralaminar glutamatergic connections in deep layers (Cauller et al., 1998; Eccles, 1984). In the case of inhibitory interneurons, electrical activity propagates through a syncytium of electrically coupled cells (Pelkey et al., 2017).

When electrical synapses are blocked the circuit is different. Experiments with pharmacologically isolated networks of inhibitory interneurons and computer model simulations have shown that these interneuronal networks can generate synchronized gamma-band oscillations, requiring only synaptic inhibition and gap junctional coupling to be intact (Deans et al., 2001; Hormuzdi et al., 2001; Traub et al., 2003). In computational models, these electrical synapses were positioned either near the PV+ interneuron cell body (soma), on the distal dendrites, or axons. Regardless of their position, electrical synapses between PV+ interneurons increase the synchrony index for both dendrite- and soma-driven networks (Kriener et al., 2022). In a 100-neuron model network, theta-nested high frequency oscillations (similar to the putative interneuronal network gamma oscillations observed experimentally in the optogenetically-driven PV-ChR2 mice), were achieved when the model gap junctions were set to contribute a significant fraction of the PV+ interneuron input resistance (Via et al., 2022). Although, connexin isoforms Cx45 and Cx57, were found to be expressed in neurons and detected in the structure of gap junction, Cx36 is the most abundantly detected neuronal connexin in the mammalian brain (Nagy and Rash, 2017). To determine the role of electrical synapses, researchers constructed mice expressing histochemical reporters (beta-galactosidase) in place of the gap junction protein Cx36. In stained histological sections, the reporter beta-galactosidase was found predominantly on the somatostatin and PV+ interneurons suggesting that Cx36 is expressed largely by inhibitory interneurons. The Cx36 knockout mice were dramatically deficient in electrical synapses; the input resistance of interneurons was significantly higher; and they produced weaker and more spatially restricted (drug-induced) electrical synchrony compared to intact mice. The authors concluded that electrical synapses containing Cx36 are critical for the generation of widespread, synchronous inhibitory activity (Deans et al., 2001; Hormuzdi et al., 2001). Furthermore, it has been suggested that Cx36 gap junctions between axons of interneurons are the most important electrical synapses for fast electrical oscillations. Namely, axonal electrical coupling is required for the gamma oscillation to occur at all, while dendritic gap junctions (Fig. 3E, *gap junction*) exert a modulatory effect (Traub et al., 2003).

However, each great simple rule such as “*gap junctions improve synchrony*” has a twist. One group performed paired recordings in Cx36 knockout mice and found that all fast-spiking interneuron pairs tested (*n* = 15/15) lacked electrical coupling. Furthermore, they did not detect any significant difference in the average amplitude of spike synchrony between simultaneously recorded pairs of fast-spiking interneurons, or IPSC synchrony between neighboring pyramidal cells, in Cx36 knockout brain slices versus normal controls. The researchers concluded that electrical coupling between fast-spiking interneurons is not essential to the generation of tight spike synchrony between these GABAergic neurons (Salkoff et al., 2015).

In summary, several facts suggest that gap junctions are an essential adaptation of PV+ interneurons: [i] abundant in PV+ interneurons and virtually non-existent in more numerous PCs, [ii] preferential electrical coupling with another PV+ interneuron and not with non-PV+ cell types; [iii] connexin KO animals show deficiencies in generation of fast oscillations; and [iv] computational modeling data show that removal of electrical synapses negatively impacts the generation of fast cortical oscillations.

## 4 Prospects

Neocortical PV+ interneurons in vivo are very sensitive to the activation of sensory inputs (Jones et al., 2000; Swadlow, 2003) and they discharge with a wide range of frequencies. Very brief APs of PV+ interneurons (0.4 ms halfwidth) and occasional high frequency outbursts of AP firing (e.g., 200 Hz) render this particular cell type difficult to explore physiologically using standard calcium imaging (Dana et al., 2014; Huang et al., 2021). Voltage imaging (Antic et al., 2016) is better suited for studying the physiology of cortical PV+ interneurons because it can address: [i] voltage instead of cytoplasmic concentration of calcium; [ii] slope of the rising phase of depolarization; [iii] duration of depolarization, [iv] slope of the decaying phase; and [v] temporal precision of the voltage peak in relation to an ongoing LFP. Genetically encoded fluorescent voltage indicators (GEVIs) are well suited to reveal fast interactions between targeted cell populations (cell-type specific recordings), on a millisecond scale (Knopfel and Song, 2019; Storace et al., 2016). The GEVI imaging technology has recently moved from the *slow-spiking pyramidal cell arena* into the *fast-spiking interneuron arena* (Evans et al., 2023; Fan et al., 2020b; Kannan et al., 2022). For example, green and red voltage sensors, Ace-mNeon2 and VARNAM2, and their reverse response-polarity variants pAce and pAceR, had enabled 1-kHz voltage recordings from >50 spiking neurons per field of view in awake mice. Brain state-dependent antagonism can be studied between two classes of neocortical interneurons: [i] somatostatin-expressing (SST^+^) and [ii] vasoactive intestinal peptide-expressing (VIP^+^). Spiking activity of individual interneurons can be acquired in parallel to hippocampal LFPs, for the purpose of studying cell-ensembles and their relations to underlying brain states (Kannan et al., 2022). Combination of two or three mutually compatible fluorescent indicators of voltage (GEVIs) with LFP or multi-electrode array recordings, would empower investigations of the dynamic interplay between PV+ interneurons (fast & short-lived oscillations) and other cortical neurons (slow and long-lived oscillations) at single-spike resolution, while imaging individual neurons longitudinally across all behavioral phases (Banerjee et al., 2020; Lagler et al., 2016). Optogenetic and chemogenetic activation or silencing of PV+ interneurons combined with behavioral tests and miniature microscope technology (Antonoudiou et al., 2020; Chamberlin et al., 2023; Jacob et al., 2018; Kim and Schnitzer, 2022; Sohal et al., 2009) may further probe the integrity of the PV+ circuit. The use of in vivo two photon imaging technology can be combined with GEVIs (Chamberland et al., 2017; Evans et al., 2023; Villette et al., 2019) and can be potentially used to characterize synaptic integration in individual PV interneurons (Francavilla et al., 2019; Judak et al., 2022; Roome and Kuhn, 2018). Recent viral strategies to target PV cells independent of the cre-lox strategies (Pouchelon et al., 2022; Vormstein-Schneider et al., 2020) would further enhance our knowledge about the PV+ cell subtypes, their structures, and functions.

However, a successful acquisition of neurophysiological data of excellent quality (large number of simultaneously recorded interneurons at high spatio-temporal resolution) in behaving animals, may fail to reveal any interesting conclusions, unless these state-of-the-art experimental data are paired with large-scale computational modeling (Borgers et al., 2012; Dura-Bernal et al., 2019; Traub et al., 1996; Wang and Buzsaki, 1996) and artificial intelligence (Fan et al., 2020a; Gholipour, 2022).

## Author's contribution

Srdjan Antic conceived the manuscript and figures. Katarina Milicevic and Brianna Barbeau produced experimental data for Fig. 2. Katarina Milicevic, Brianna Barbeau, Darko Lovic, Aayushi Patel and Violetta Ivanova edited and commented on the manuscript and figures. All authors: performed writing, reviewing, and editing.

## CRediT authorship contribution statement

**Katarina D. Milicevic:** Investigation, Writing – original draft. **Brianna L. Barbeau:** Investigation, Writing – review & editing. **Darko D. Lovic:** Writing – review & editing. **Aayushi A. Patel:** Writing – review & editing. **Violetta O. Ivanova:** Writing – review & editing. **Srdjan D. Antic:** Conceptualization, Funding acquisition, Supervision, Writing – review & editing.

## Declaration of competing interest

Katarina D. Milicevic, Brianna L. Barbeau, Darko D. Lovic, Aayushi A. Patel, Violetta O. Ivanova and Srdjan D. Antic, declare that they have no known competing financial interests or personal relationships that could have appeared to influence the work reported in this paper.

## Data Availability

No data was used for the research described in the article.
