# Supplementary material for: Physiological features of parvalbumin-expressing GABAergic interneurons contributing to high-frequency oscillations in the cerebral cortex
Source: Curr Res Neurobiol. 2023 Dec 16;6:100121. doi: 10.1016/j.crneur.2023.100121 (PMC11015061; doi:10.1016/j.crneur.2023.100121)
Supplement: Multimedia component 1 [file mmc1.pdf]

Peer Review Overview

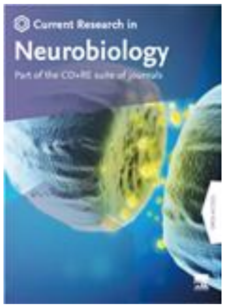

Manuscript Title: Physiological Features of Parvalbumin-Expressing GABAergic Interneurons Contributing to High-Frequency Oscillations in the Cerebral Cortex

Received	Mar 28, 2023
1st Decision	Jul 23, 2023
1st Revision Submitted	Aug 29, 2023
2nd Decision	Oct 26, 2023
2nd Revision Submitted	Nov 13, 2023
Accepted	Dec 01, 2023

1st Decision letter

Reference: CRNEUR-D-23-00031

Title: Physiological Features of Parvalbumin-Expressing GABAergic Interneurons Contributing to High-Frequency Oscillations in the Cerebral Cortex
Journal: Current Research in Neurobiology

Dear Associate Professor Antic,

Thank you for submitting your manuscript to Current Research in Neurobiology.

I have completed my evaluation of your manuscript. The reviewers recommend reconsideration of your manuscript following revision. I invite you to resubmit your manuscript after addressing the comments below. Please resubmit your revised manuscript by Sep 06, 2023.

When revising your manuscript, please consider all issues mentioned in the reviewers' comments carefully: please outline every change made in response to their comments and provide suitable rebuttals for any comments not addressed. Please note that your revised submission may need to be re-reviewed.

Current Research in Neurobiology values your contribution and I look forward to receiving your revised manuscript.

Kind regards,

Abhishek Banerjee
Associate Editor
Current Research in Neurobiology

Comments from Editors and Reviewers:

Reviewer #1:

Milicevic et al provide a review of one of the most abundant and well-studied of GABAergic interneurons in the brain. There is a vast literature on this interneuron subtype and, as one can appreciate, to provide a comprehensive yet clear overview is not an easy task. In the current review, the authors specifically focus on morpho-physiological aspects of parvalbumin interneurons. Together these features are pertinent to their computational functionality such as generations of network rhythms and hence together shape their role in neural circuits. The review is in general well-written but at times can be quite hard to follow with issues of logical flow and repetition of concepts. Nevertheless, the review will be of use for not only beginners to the field but also to the seasoned researcher – rather than to digest as a whole, but perhaps to focus on specific aspects of PV interneurons that are of particular interest to each reader. I have a series of comments for consideration.

Overall/general comments:

1. The authors employ numerous words/phrases in quotation marks. I understand this is stylistic but results in the review having a rather colloquial nature in many parts. I suggest perhaps limiting these throughout the review.
2. A number of sections including the "Suppression of the out-of context cells" and "Gamma Band" sections are a difficult read particularly for a novice audience. I am not sure what the best course of action is here - perhaps by simplifying the message, if possible. On a similar note, the review at times suffers from issues of logical flow. For example, in section 3-06 the authors describe various properties resulting in a propensity for linear dendritic synaptic integration. This is then followed by a section titled "Linear Dendritic integration of afferent inputs". Similarly in Section A the authors have sections "cortical inputs intercepted by PV+ interneurons" which is then followed by a section 3-02 on afferent excitatory inputs – this is a repetition and could be consolidated.
3. Please consider including additional statements to literature concerning investigation into parvalbumin interneuron in non-human primates and humans as already included for the laminar distribution of cortical PVs and the Szegedi manuscripts. e.g. other studies that could be mentioned are Povysheva et al., 2008 J. Neurophysiol; Gonzalez-Burgos et al., 2005 J Neurophsyiol; Wang et al., 2016; Front Cell Neurosci; Szegedi et al., 2023 PLOS Biol. Also note in the myelination section the authors cite the Stedehouder et al., study but do not mention in the text that PV myelination is conserved in humans.
4. In the Prospects section an explicit mention of the use of in vivo two photon and miniature microscope technology would be welcome. These techniques are now in common use in conjunction with the various calcium/voltage dyes and optogenetic approaches discussed by the authors. Also reference to recent viral strategies to target PV cells independent of the cre-lox strategies described (Vormsten-Schnieder et al., 2020 Nat Neurosci; Pouchelon et al., 2022 Cell Reports) would be welcome.

Specific Comments:

1. Highlights
(a) Please reconsider the terminology "intercept". This conjures the notion that PV cells block information transfer into and out of the cortex. Perhaps sculpt is more appropriate.
(b) "carve" – again this is rather colloquial in nature and maybe another terminology should be used e.g. dictate, control
(c) Please rephrase the myelination statement. Although it is clear throughout the review main text, the way that it is written in the highlights may lead the novice reader to think that only PV-interneuron axons are myelinated over any neuronal subtype (including glutamatergic) within the CNS. The authors need to rephrase to make it clear it is only out of the interneuron subclasses that PV axons are myelinated. I would also suggest adding the term "the vast majority" rather than "only".
(d) Authors need to be careful here regarding the summary of the site of PV outputs onto PCs that serve to efficiently control the timing and success of PC spiking. Of course, a subset of PVs (chandelier cells) specifically target the axon initial segment but the perisomatic targeting PVs (which are arguably the bigger subset of cortical PVs) also effectively perform this function as discussed in the main text.

2. Abstract
(a) "we discuss two groups" – perhaps change "groups" to "aspects".
(b) "Given the link between…." I would reconsider altering the final sentence of the abstract. Of course, there is much to learn but PV cells are arguably one of the most studied IN subclass in CNS circuitry. I would mention the possible great strides that will be made due to relatively recent advent of scientific tools such as transgenic mice, viral strategies to target PV cells and the ability to monitor PV cells in vivo imaging during behavior will aid in defining role of PV cells in the normal/pathophysiological CNS.

3. Introduction
(a) The authors mention that the review is tailored towards those relatively new to the subject of parvalbumin interneurons "(e.g. 1st year PhD students)". I think the review would be of interest to those researchers both new and experienced alike. I would therefore suggest removing this statement suggesting otherwise.

4. Main Text
(b) In the interest of logical flow, the authors could include a sentence somewhere in section 1 "Unnecessary spikes" section introducing/explaining the concept of feed-forward inhibition. This is a key aspect in spike control and timing of PCs, particular those relating to thalamic recruitment of PV INs in sensory cortices for instance as the authors discuss later in the review.
(c) As the authors point out, one of the key features of parvalbumin cells is a rapid kinetic of their synaptic excitatory inputs. However, the section regarding this is rather sparse despite that this is a very important aspect of the temporal precision of PV interneuron recruitment. Thus, although the review is long, I do believe that the authors should include a sentence or two describing the molecular underpinnings of this rapid synaptic input. e.g. small NMDA/AMPA ratios, GluA2-lacking/GluA4 containing calcium-permeable receptors.
(d) In the context inhibition section, the authors should explicitly state that "135 degree"and "225 degree" relate to visual drifting bar stimuli.
(e) Section 3.02 - Do the authors mean to use the term "didactic"? Also, the distinction between "local" and "faraway" seems arbitrary and requires further clarification? Perhaps splitting into intracortical/ipsi and contralateral and extracortical would be more appropriate. Furthermore, the authors mention thalamic projections in their "local" input section.

(f) In the section discussing the reversed gradient of input resistance the sole citation is that from the Jonas laboratory (Norenberg et al). This study focusses on PV basket cells in the dentate gyrus of hippocampus. Have similar studies been performed in cortex? If so, this should be included in the review. If not, the authors should state that this property although likely conserved across brain regions remains to be empirically ascertained for cortical PVs.
(g) 3.16. Could the authors expand in a few sentences where gap junctions are present between PV INs, Is it true that most are perisomatic? Unless I am mistaken at least from PV stains of soma in cortical regions very few soma are in close enough position for this to be a predominant region for gap junctions – thus are the majority of gap junction on dendrites or axonal compartments?

5. Figures
(a) To demonstrate the laminar positioning of cortical PV interneurons the authors include data from their lab using PV-ASAP2 mouse. For clarity the authors should mention that this is a genetically encoded voltage dye. Is this data published? If so, the citation needs to be included in the legend/reference section of the figure.

Minor comments/typos
(a) Correct "tunning" in the "suppression of the baseline firing – blanket inhibition" section.
(b) Section 3.01 – Please "correct" the italic paragraph that appears in this section. It seems out of place.
(c) Correct "bring"to "bringing" in the "suppression of the out-of-context cells" section
(d) "TAU" does not require capitalization throughout the manuscript.

1st Author Response Letter

Response to comments from Editors and Reviewers:

Dear Editor Banerjee,
We are impressed with the quality of the critiques. The reviewer took an enormous amount of time and effort to identify weaknesses, and more importantly to offer excellent solutions. We are impressed with the reviewer's command of the literature and attention to detail. We enjoyed revising the manuscript, as we see that its quality improved with each individual critique being addressed.
We hope that the current review article will be of use for not only beginners to the field but also to the seasoned researchers. As pointed out by the reviewer, rather than to digest as a whole, readers can easily navigate and focus on specific aspects of PV interneurons that are of particular interest to each reader, because our text is comprised of units conveniently listed in boxes (Box 1 & Box 2), but also laid out in the form of a cartoon (Fig. 1E).
The critiques were accurate and very constructive (blue font below). We addressed each and every issue brought by the reviewer and in more than 90% of instances, we were able to fully comply with the reviewer's instructions. Below we outline in a cover letter every change made in response to the reviewer's comments, and we provide suitable rebuttals for one comment not addressed (Peter Jonas Lab: small NMDA/AMPA ratios and calcium-permeable AMPA receptors). In the body of the manuscript, all changes are marked by red font.

Comments from Reviewer 1:

Overall/general comments:

1. The authors employ numerous words/phrases in quotation marks. I understand this is stylistic but results in the review having a rather colloquial nature in many parts. I suggest perhaps limiting these throughout the review.

We thank the reviewer for this well-placed critique. Indeed, many of our phrases could be interpreted as colloquial in nature. We went back to the submitted version of the manuscript and identified 83 points in which we use words and phrases in quotation marks. Clearly, too many. At ~80% of those instances, we removed quotation marks completely or modified the words, without affecting the meaning of the sentence.

2a. A number of sections including the "Suppression of the out-of context cells" and "Gamma Band" sections are a difficult read particularly for a novice audience. I am notsure what the best course of action is here - perhaps by simplifying the message, if possible.

We revised text in two sections titled "Suppression of the out-of-context cells – context inhibition" and "Fast Electrical Oscillation - Gamma Band – Temporal Code". We modified these texts to make them suitable for novice audience. In the revised version of the manuscript, these new changes are marked by red font.

2b. On a similar note, the review at times suffers from issues of logical flow. For example, in section 3-06 the authors describe various properties resulting in a propensity for linear dendritic synaptic integration. This is then followed by a section titled "Linear Dendritic integration of afferent inputs". Similarly in Section A the authors have sections "cortical inputs intercepted by PV+ interneurons" which is then followed by a section 3-02 on afferent excitatory inputs – this is a repetition and could be consolidated.

Section 3-06 prepares the readers for section 3-07.

Specifically, in section 3-06, subtitle [*] "Reversed gradient of Rm along the somatodendritic axis". The topics are: [i] local input resistance (Rm), [ii] Rm affects both the amplitude and temporal dynamics of the local voltage waveforms, [iii] fast time courses of synaptic potentials arriving at the soma, and [iv] weak RC filtering. In section 3-06, we also have a subtitle describing a set of biophysical features dubbed "Active membrane properties that shape the EPSPs in PV+ interneurons". Specifically, these topics include: [v] High-threshold fast-acting K+ current, which is responsible for slimming the EPSPs waveforms, [vi] amplitude compression imposed on dendritic voltage transients, and [vii] shallow input-output (I-O) function. None of these topics is directly related to "linear dendric integration". Furthermore, in section 3-06 we also describe glutamate-mediated nonlinear responses, which are opposite from the linear dendritic integration described in section 3-07. In the revised version of the manuscript, we emphasized the part of the Section 3-06 devoted to the discussion of nonlinear dendritic properties; new subtitle: "Nonlinear dendritic properties in PV+ interneurons". These three parts of 3-06, are then followed by a section 3-07 titled: "Linear Dendritic integration of afferent inputs".

3. Please consider including additional statements to literature concerning investigation into parvalbumin interneuron in non-human primates and humans as already included for the laminar distribution of cortical PVs and the Szegedi manuscripts. e.g. other studies that could be mentioned are Povysheva et al., 2008 J. Neurophysiol; Gonzalez-Burgos et al., 2005 J Neurophsyiol; Wang et al., 2016; Front Cell Neurosci; Szegedi et al., 2023 PLOS Biol. Also note in the myelination section the authors cite the Stedehouder et al., study but do not mention in the text that PV myelination is conserved in humans.

3a

We included additional statements to literature concerning investigation into parvalbumin interneuron in non-human primates and humans. New text is formatted red, but also shown below for quick check:

>> The cell body diameter, total dendritic length, and horizontal and vertical spans of the axonal arbor of PV cells were similar in monkeys and rats, although the monkey basket cells appeared to be more excitable (Gonzalez-Burgos et al., 2005; Povysheva et al., 2008). In rodents, monkeys and humans, the maximal firing frequency of FS neurons increases with the species hierarchy, and varies based on the cortical area and age, with mean firing frequency often exceeding 400 Hz (Wang et al., 2016). The cell body of human neocortical PV+ basket interneuron amply express HCN channels, which shorten the lag between excitatory postsynaptic potentials and action potential generation in human, whereas these channels are sparse at the rodent basket cell soma membrane (Szegedi et al., 2023). <<

3b

In the revised text, we now state that PV myelination is conserved in humans:

>> PV+ IN myelination is conserved in human. Using acutely resected cortex from human patients, Stedehouder et al (2017) showed that all of the human PV+ interneurons examined (8/8 cells), but none of the non-fast-spiking cells (0/3 cells), exhibited myelinated axons. Similar to their finding in mice, PV+ interneuron myelination in human neocortex was biased towards proximal axonal segments (Stedehouder et al., 2017). Interestingly, mouse and human neocortical interneurons follow similar morphological rules guiding the topography of axonal myelination. The topography of myelination along individual PV+ axons is strongly predicted by the joint combination of axonal diameter and interbranch distance (Stedehouder et al., 2019). The conserved feature of both the mouse and human brain islikely a consequence of the evolutionary pressure to conserve efficient mechanism to ensure that a high proportion of action potentials pass through myelinated segments, thereby potentially enhancing conduction velocity, fidelity, and metabolic support. <<

4. In the Prospects section an explicit mention of the use of in vivo two photon and miniature microscope technology would be welcome. These techniques are now in common use in conjunction with the various calcium/voltage dyes and optogenetic approaches discussed by the authors. Also reference to recent viral strategies to target PV cells independent of the cre-lox strategies described (Vormsten-Schnieder et al., 2020 Nat Neurosci; Pouchelon et al., 2022 Cell Reports) would be welcome.

In the Prospects section, we now make an explicit mention of the use of in vivo two photon and miniature microscope technology. In addition, we include the new viral strategy for labeling PV interneurons. The new text is marked by red font in the manuscript. Here we include a copy of the new text:

>> Optogenetic and chemogenetic activation or silencing of PV+ interneurons combined with behavioral tests and miniature microscope technology (Antonoudiou et al., 2020; Chamberlin et al., 2023; Jacob et al., 2018; Kim and Schnitzer, 2022; Sohal et al., 2009) may further probe the integrity of the PV+ circuit. The use of in vivo two photon imaging technology can be combined with GEVIs (Chamberland et al., 2017; Villette et al., 2019) and can be potentially used to characterize synaptic integration in individual PV interneurons (Francavilla et al., 2019; Judak et al., 2022; Roome and Kuhn, 2018). Recent viral strategies to target PV cells independent of the cre-lox strategies (Pouchelon et al., 2022; Vormstein-Schneider et al., 2020) would further enhance our knowledge about the PV+ cell subtypes, their structures and functions. <<

Specific Comments:

1. Highlights

(a) Please reconsider the terminology "intercept". This conjures the notion that PV cells block information transfer into and out of the cortex. Perhaps sculpt is more appropriate.

We will use "sculpt" instead of "carve" later, see critique (b).

In our view, the term "intercept" is not 100% synonymous with a block. For example, police officers intercept bad drivers along the highway, issue fines / warnings, and then they let these drivers go their ways.

Our intention is to highlight the notion that information can neither enter nor leave the neocortex without strong engagement of fast PV+ cell-mediated inhibition. The new text is formatted red in the manuscript file, but also shown below for a quick check:

"PV+ interneurons are positioned in cortical layers 2–6, to intercept and filter cortical input into L4 & L5 (thalamic projections), and then intercept and filter cortical outputs – from L2 & L3 to other cortical areas (cortico-cortical projections)".

(b) "carve" – again this is rather colloquial in nature and maybe another terminology should be used e.g. dictate, control

We agree with the reviewer. The new text is formatted red in the manuscript file, but also shown below for a quick check:

>> PV+ interneurons use fast spiking to outpace other excitable cells in the cortex and sculpt the ongoing electrical rhythm. <<

(c) Please rephrase the myelination statement. Although it is clear throughout the review main text, the way that it is written in the highlights may lead the novice reader to think that only PV-interneuron axons are myelinated over any neuronal subtype (including glutamatergic) within the CNS. The authors need to rephrase to make it clear it is only out of the interneuron subclassesthat PV axons are myelinated. I would also suggest adding the term "the vast majority" rather than "only".

Good point. Per reviewer's critique, the new text is shown below:

>> The vast majority of cortical excitatory pyramidal cells have myelin wraps around their axons. However, among the cortical inhibitory interneurons, only the PV+ subclass has myelinated axons, which are used to secure fast spiking in the initial axon segment (first), and then shorten time delays between the PV+ interneuron action potential and synaptic releases of GABA at the PV+ axon terminals (second). <<

Please note that later in text, we have previously stated:

>> Among all cortical inhibitory GABAergic interneurons, only the PV+ subclass possesses a myelinated axon (Stedehouder et al., 2017). <<

(d) Authors need to be careful here regarding the summary of the site of PV outputs onto PCs that serve to efficiently control the timing and success of PC spiking. Of course, a subset of PVs (chandelier cells) specifically target the axon initial segment but the perisomatic targeting PVs (which are arguably the bigger subset of cortical PVs) also effectively perform this function as discussed in the main text.

Good point. Indeed, our text in the highlights implied that PV+ synaptic contacts are exclusively made on axons. We fixed this problem with the following modifications:

>> Strategic positioning of the PV+ inhibitory synapses onto the pyramidal neuron's cell body and axon initial segment is used to robustly obliterate spiking in cortical pyramidal cells, or paradoxically excite cortical pyramidal neurons, with a near 100% success rate. <<

2. Abstract

(a) "we discuss two groups" – perhaps change "groups" to "aspects".

The revised text is shown below for a quick check.

>> In this review, we discuss two groups/aspects of fundamental properties ... <<

(b) "Given the link between…." I would reconsider altering the final sentence of the abstract. Of course, there is much to learn but PV cells are arguably one of the most studied IN subclass in CNS circuitry. I would mention the possible great strides that will be made due to relatively recent advent of scientific tools such as transgenic mice, viral strategies to target PV cells and the ability to monitor PV cells in vivo imaging during behavior will aid in defining role of PV cells in the normal/pathophysiological CNS.

Good point. The new Abstract text is show below for a quick check:

>> Recent advent of scientific tools, such as viral strategies to target PV cells and the ability to monitor PV cells in vivo imaging during behavior, will aid in defining role of PV cells in the CNS. Given the link between PV+ interneurons and cognition, in the future, it would be useful to carry out physiological recordingsin the PV+ cell type selectively and characterize if and how psychiatric and neurological diseases affect initiation and propagation of electrical signals in this cortical sub- circuit. Voltage imaging may allow fast recordings of electrical signals from many PV+ interneurons simultaneously. <<

3. Introduction

(a) The authors mention that the review is tailored towards those relatively new to the subject of parvalbumin interneurons "(e.g. 1st year PhD students)". I think the review would be of interest to those researchers both new and experienced alike. I would therefore suggest removing this statement suggesting otherwise.

We agree. This sentence has been removed.

4. Main Text

(b) In the interest of logical flow, the authors could include a sentence somewhere in section 1 "Unnecessary spikes" section introducing/explaining the concept of feed-forward inhibition. This is a key aspect in spike control and timing of PCs, particular those relating to thalamic recruitment of PV INs in sensory cortices for instance as the authors discuss later in the review.

We agree. The revised text is show below for a quick check:

>> Feedforward inhibition is the most basic cortical circuit mechanism, which critically depends on the intact function of PV+ INs (Tremblay et al., 2016). In feedforward inhibition, an external source (such as thalamus or neighboring cortex) makes excitatory synapses onto both local PCs and PV+ INs (Buzsaki, 1984). The PV+ INs respond faster than PCs, thus allowing PV+ IN to inhibit many PC neighbors (Swadlow et al., 1998). Feedforward inhibition is thus a circuit mechanism designed to restrict activity to a small

number of cortical PCs, depending on their specialization (e.g. receptive field, temporal window of opportunity) (Isaacson and Scanziani, 2011; Swadlow, 2003; Tremblay et al., 2016). <<

(c) As the authors point out, one of the key features of parvalbumin cells is a rapid kinetic of their synaptic excitatory inputs. However, the section regarding this is rather sparse despite that this is a very important aspect of the temporal precision of PV interneuron recruitment. Thus, although the review is long, I do believe that the authors should include a sentence or two describing the molecular underpinnings of this rapid synaptic input. e.g. small NMDA/AMPA ratios, GluA2-lacking/GluA4 containing calcium-permeable receptors.

This is a valid suggestion, however small NMDA/AMPA ratios and GluA2-lacking/GluA4 containing calcium-permeable receptors have been already thoroughly described in Peter Jonas publications (review articles). In our paper, this topic would require a number of complex sentences and a figure panel. We discussed this suggestion and decided not to include descriptions of the molecular underpinnings of this rapid synaptic input in our already very long article. In the revised manuscript, we direct readers in the direction of glutamate receptors with the following text.

>> In the cortex and hippocampus, temporal dynamics of AMPA-receptor-mediated excitatory postsynaptic current influences the rise time of an EPSP. It is thought that AMPA receptors on hippocampal FS PV+ cells are made of AMPA subunit isoforms especially adapted to produce fast rising postsynaptic currents(discussed in (Jonas et al., 2004)). Neocortical FS PV+ cells may indeed employ fast-acting AMPA receptors, as their EPSP voltage waveforms are also very rapid – fast rising.

(d) In the context inhibition section, the authors should explicitly state that “135 degree” and “225 degree” relate to visual drifting bar stimuli.

We agree. The revised text is show below for a quick check:

>> For example, PV+ cells sharply tuned to 135 deg (visual drifting bar) are expected to strongly suppress PCs that are trained to respond to 225 deg (Nowak et al., 2008). <<

(e) Section 3.02 - Do the authors mean to use the term “didactic”? Also, the distinction between “local” and “faraway” seems arbitrary and requires further clarification? Perhaps splitting into intracortical/ipsi and contralateral and extracortical would be more appropriate. Furthermore, the authors mention thalamic projections in their “local” input section.

In Section 3.02 – We indeed meant to use the term “didactic”. In our text, we organized synaptic afferents in a simple way: Local and Faraway. Our textual organization of the afferents is combined with the graphical organization (Fig. 2BC). Here, “didactic” refers to our presentation method that follows an organized and simplified approach, or educational style, to deliver information to novices. Intracortical inputs can be both local and faraway – local if coming from the same cortical column – faraway if coming from a neighboring or remote cortical area. We decided to use Local and Faraway, as our didactic approach is clearly delineated in Fig. 2BC.

We agree with the reviewer that we created confusion by mentioning thalamic afferents in the paragraph titled “Local”. To fix this error we completely reorganized section 3.02. Here is the new text for a quick check up:

>> For didactic purposes, we divide afferent excitatory inputs to PV+ cells into two groups: Local and Faraway.

From Local Cells. The local group is made of cells in the same cortical column (or the neighboring column) (Dantzker and Callaway, 2000), including inhibitory fast-spiking (FS) PV+ interneurons, inhibitory non-FS interneurons, and excitatory PCs (Fig. 2B).

From Faraway Regions. Remote brain regions also supply excitatory glutamatergic inputs onto neocortical PV+ interneurons, and these include: the thalamus, ipsilateral cortex, and contralateral cortex (Fig. 2C). The most abundant faraway afferents are from the ipsilateral cortex, and they can roughly be divided into feedforward (from lower to higher processing hierarchy) and feedback (from higher to lower information processing hierarchy). Interestingly the feedforward cortico-cortical contacts onto PV+ interneurons are: [i] significantly larger in diameter, [ii] contain more mitochondria, and [iii] contain more docked synaptic vesicles, compared to the feedback cortico-cortical contacts (Gonchar and Burkhalter, 1999).

In Fig. 2, the size of an arrow suggests the relative number of inputs contributed by that cell class. For example, FS PV+ cells provide the greatest fraction of local afferents onto the PV+ cell dendrites(Fig. 2B, turquoise arrow), and these contacts are inhibitory in nature. Two kinds of local cells (cortical L5-L6 pyramidal neurons and L4 stellate cells) and faraway cells (thalamic projection neurons) provide excitatory glutamatergic synapses onto PV+ interneurons (Bagnall et al., 2011; Gonchar and Burkhalter, 1999; Komlosi et al., 2012; Molnar et al., 2016; Swadlow et al., 1998). In PV+ L4 basket cells of the cat visual cortex, equal numbers of synapses are provided by the L6 PCs (~40%) and the spiny stellate cells(~40%), whereasthe faraway thalamic afferents contribute only ~15%. As much as 79% of the inhibitory GABAergic synapses originate from other L4 basket cells (Ahmed et al., 1997). Localspiny stellate and faraway thalamic synapses are preferentially located on the soma and proximal dendrites of PV+ interneurons, while faraway cortico-cortical projections are rather located on the distal dendrites (Ahmed et al., 1997; Bagnall et al., 2011; Freund et al., 1985). <<

(f) In the section discussing the reversed gradient of input resistance the sole citation is that from the Jonas laboratory (Norenberg et al). This study focusses on PV basket cells in the dentate gyrus of hippocampus. Have similar studies been performed in cortex? If so, this should be included in the review. If not, the authors should state that this property although likely conserved across brain regions remains to be empirically ascertained for cortical PVs.

Good point. Unfortunately, the Jonaslab isthe only one who found thisin PV+ interneurons. In the revised version we include the following excellent sentence (we took directly from the reviewer):

>> Dual (soma & dendrite) patch electrode recordings combined with computational modeling indicate that specific membrane resistance (Rm) in hippocampal PV+ interneurons is markedly nonuniform, with a 40-fold higher value in the axon, and a 10-fold higher value in the distal dendrites, than in the proximal dendrites and the soma (Norenberg et al., 2010). This property although likely conserved across brain regions remains to be empirically ascertained for cortical PV cells. <<

(g) 3.16. Could the authors expand in a few sentences where gap junctions are present between PV INs, Isit true that most are perisomatic? UnlessI am mistaken at least from PV stains ofsoma in cortical regions very few soma are in close enough position for this to be a predominant region for gap junctions – thus are the majority of gap junction on dendrites or axonal compartments?

The new addition to the gap junction section is show below for a quick check:

>> Parvalbumin (PV)-positive interneurons form dendritic gap junctions with one another, with the majority (~85%) of dendritic gap junctions within 75 µm from the cell body (Shigematsu et al., 2019). The overwhelming majority of PV-to-PV electrical synapses are located on cell body and near the cell body (Galarreta and Hestrin, 1999; Gibson et al., 1999; Tamas et al., 2000). Histological evidence for axonal gap junctions in the cortex is not as strong as for dendritic gap junctions, but electrophysiological studies and computer simulations have provided evidence for their existence in the neocortex (Traub et al., 2001). Interestingly, PV-PV axonal gap junctions operate in amygdala (Muller et al., 2005). <<

5. Figures

(a) To demonstrate the laminar positioning of cortical PV interneuronsthe authorsinclude data from their lab using PV-ASAP2 mouse. For clarity the authors should mention that this is a genetically encoded voltage dye. Is this data published? If so, the citation needs to be included in the legend/reference section of the figure.

This is our unpublished data. We now state this in the figure caption (Fig. 2A-i).

Minor comments/typos

(a) Correct “tunning” in the “suppression of the baseline firing – blanket inhibition” section.
Done.

(b) Section 3.01 – Please “correct” the italic paragraph that appears in this section. It seems out of place.

The italic paragraph is designed to stand out in the page, akin to a Text Box one often finds in review articles. Text Box is a visually distinct section that is used to highlight and present specific information within the main body of the article. It is typically set apart from the regular text by using a shaded background, a border, or a different font style (e.g. italic). Text boxes are often used to emphasize key points, provide additional context, present case studies or examples, or discuss related topics that are relevant to the main content of the article. Here, we use it to emphasize a key point. In the final PDF, we hope to have a shaded background, as shown in the revised manuscript.

(c) Correct “bring” to “bringing” in the “suppression of the out-of-context cells” section

Done

(d) “TAU” does not require capitalization throughout the manuscript.

Throughout the manuscript we replaced TAU with tau.

With the numerous changes incorporated into the text, we feel that our manuscript has been notably improved, and perhaps, could now be considered for publication?

2nd Decision letter

Reference: CRNEUR-D-23-00031

Title: Physiological Features of Parvalbumin-Expressing GABAergic Interneurons Contributing to High-Frequency Oscillations in the Cerebral Cortex
Journal: Current Research in Neurobiology

Dear Associate Professor Antic,

Thank you for submitting your manuscript to Current Research in Neurobiology.

I have completed my evaluation of your manuscript. The reviewers recommend reconsideration of your manuscript following minor revision and modification. I invite you to resubmit your manuscript after addressing the comments below. Please resubmit your revised manuscript by Nov 25, 2023.

When revising your manuscript, please consider all issues mentioned in the reviewers' comments carefully: please outline every change made in response to their comments and provide suitable rebuttals for any comments not addressed. Please note that your revised submission may need to be re-reviewed.

Current Research in Neurobiology values your contribution and I look forward to receiving your revised manuscript.

Kind regards,

Abhishek Banerjee
Associate Editor
Current Research in Neurobiology

Comments from Editors and Reviewers:

Reviewer #2:

This is a comprehensive and interesting review on parvalbumin-expressing GABAergic neurons in relation to gamma oscillations within the mammalian neocortex. To improve the accessibility and readability, I have some suggestions.
General points:
As I understand, the aim is to make it understandable to students in early stages of their careers. In my view, there is a risk that introducing new terms may mislead them; using familiar and precise scientific terms will increase the understanding and clarify what is such a diverse and complex topic. But I appreciate this also relates to the authors (quite rightly) wishing to use a more engaging writing style, so the balance has to be right. For example, 'Before Axon' and 'After Axon', which seems to refer to subcellular domains either side of the axon initial segment. The latter could be interpreted as postsynaptic mechanisms ('after' the presynaptic terminals of the axon). Rather, Inputs (e.g. synaptic integration) versus Outputs (e.g. neurotransmission), are precise and familiar terms. Similarly, 'faraway'

versus 'local' inputs. I am not sure about the term 'faraway' as most readers would already understand that subcortical inputs to a particular cortical region are not local, and cortico-cortical inputs are by definition inputs from other cortical areas.
It is common for many reviews and articles to mix terms such as type, subtype, class, subclass. It would help to be consistent, e.g. PV+ neurons form a subpopulation of cortical GABAergic neurons. Within this subpopulation, there are several types, with similar features within each cortical area. In the hippocampus, there are several types of PV interneurons that also express somatostatin and/or NPY, which is different to the isocortical PV cells.
Despite the first sentence of the Introduction, it is not always clear if the review is about neocortical PV cells or all cortical PV cells (including the hippocampus). In most cases, the cortical area is specified, but there are several places where the specific region of cortex would help the reader assess the statement/evidence. There is a danger to generalise findings. For example, the main claim of a reversed Rm gradient (even mentioned in the abstract) is based on Norenberg et al 2010, which is from the dentate gyrus - clearly not the neocortex. The dentate gyrus is a highly specialized area and PV cells in other cortical areas may be different. Another claim is that PV neurons are the only type of myelinated interneuron. But many types of GABAergic neuron (including somatostatin-expressing neurons) in the hippocampus are myelinated, and many also have local axon and projection axons. To avoid misleading the reader, it would help to (1) specify the exact sources of the observation(s), (2) avoid making binary statements (e.g. 'no other interneuron is myelinated' should be 'myelinated axons have only been observed in PV neurons in the neocortex' - other types of interneuron may well have myelinated axons and they just have not been detected yet). Another example statement: "Information can neither enter nor leave the neocortex without the engagement of the fast PV+ mediated cortical inhibition" - evidence? One could use different terms depending on the amount of evidence or confidence ('we hypothesize', 'we speculate', 'we suggest' etc.).
Somogyi P, Freund TF, Cowey A (1982) The axo-axonic interneuron in the cerebral cortex of the rat, cat and monkey. Neuroscience 7: 2577-2607. This is the first evidence of myelination of axo-axonic cell axons.
More specific comments:
Title: High-frequency oscillations, if defined in terms of the local field potential (LFP), emerge from the summed extracellular currents flowing across the membranes of all cells, not just the PV cells (eg see Buzsaki et al Nat Rev Neurosci. 2012;13:407-20). Alternative title: Physiological Features of Parvalbumin-Expressing GABAergic Neurons Contributing to High-Frequency Oscillations in the Cerebral Cortex / Neocortex.
Public and media statement: Typo Careers. 'Physiology of' - physiological roles of? See also above point on cell types - variety of PV+ interneuron types.
If this is a review for early-career scientists, one should briefly define GABA and inhibition in general, as well as hyperpolarizing and shunting. See also recent paper on fast synaptic inhibition in vivo (Burman et al Neuron 2023 Active cortical networks promote shunting fast synaptic inhibition in vivo.
Highlights: The term 'outpace' is unclear, as are 'competing networks'. Overwhelm = overcome? PV+ interneurons inhibit other excitable (glutamatergic, or excitatory?) cells within short time windows during the ongoing electrical rhythm - all neurons are excitable.
'The vast majority of cortical excitatory [glutamatergic] pyramidal cells have myelin 'sheaths' around their axons.
'Strategic positioning of the PV+ inhibitory synapses'. 'Strategic' suggests there was a strategy, rather it is evolution/development. Are spikes obliterated or just that the membrane potential is hyperpolarized or there is shunting? Evidence for a 100% success rate?
Abstract: 'It would be useful to carry out [further] physiological recordings [of] PV+ cells selectively. See existing published studies on identified PV cells and cognition e.g. Lagler et al Neuron 2016 Divisions of

Identified Parvalbumin-Expressing Basket Cells during Working Memory-Guided Decision Making.
"The overwhelming majority of PV-to-PV electrical synapses are located on cell body and near the cell body." - meaning between a cell body and a dendrite or 2 cell bodies?
Introduction:
'Really fast and mighty strong' - not clear terms, and in relation to what other cell types and timescales?
Claim of 'never before included in a PV interneuron review article' - this is a potentially misleading statement, perhaps unnecessary. Type-2 firing is not defined. What is type-1 firing?
Minor: figures are not cited in order.
'Unnecessary spikes' and 'sparse coding' - need strong definitions here as this is subjective and dependent on behavioural state, model, anesthesia, and brain region, and also recording/imaging technique. E.g. 'sparse coding' is often referred in models where all but one whisker are trimmed, focusing on mouse somatosensory cortex. In this paragraph, again the brain region ("sensory" cortex or other cortical areas should be specified as may not apply to all cortical areas).
'Cumbersome baseline firing' - again this term is subjective, as brain operations are not normally considered cumbersome, given energy demands.
"Suppression of the baseline firing" paragraph - claim of 200um region, specify if this is a diameter or width of a cortical area, and which cortical region it refers.
"Pyramidal neuron network dynamic range" paragraph - specify which brain area and species these data were obtained.
Neuronal ensembles - see also neuronal assemblies. The use of these terms is often brain-region dependent and behavior-dependent. Suggest giving examples or specifying brain regions referring to.
"Pyramidal cells (PCs) that respond to animal-like objects appearing in the perceptual space, should not respond when plant-like objects appear" - evidence? Is this statement a speculation? 'Plant-like' object cells and animal-like object cells - If no evidence, state these as examples or hypotheses, as the cited papers seem to not relate to them.
Gamma oscillations: Further insight into different types of gamma oscillations and coupling (relevant to PV cells) see Lasztóczi B, Klausberger T. Distinct gamma oscillations in the distal dendritic fields of the dentate gyrus and the CA1 area of mouse hippocampus. Brain Struct Funct. 2017;222(7):3355-3365.
Box 1, typo glutamate receptors
Box 2, GABA 'release onto' rather than 'delivery on'?

2nd Author Response Letter

Response to comments from Editors and Reviewers:

Dear Dr. Banerjee, Thank you for reviewing our manuscript to Current Research in Neurobiology. We have completed our revision of the manuscript. The reviewers made some excellent recommendations, and we gladly adopted the great majority of their suggestions. When revising our manuscript, we considered all issues mentioned in the reviewers' comments carefully. We outlined every change made in response to their comments using RED FONT, and we provided rebuttals for the comments not addressed.

Comments from Reviewer 2:

This is a comprehensive and interesting review. I have some suggestions. We organized the list of reviewer's comments using numerical sequencing (1 – 31). Reviewer's critiques are in blue font, and the authors' responses are in black font.

1 For example, 'Before Axon' and 'After Axon', which seems to refer to subcellular domains either side of the axon initial segment. The latter could be interpreted as postsynaptic mechanisms ('after' the presynaptic terminals of the axon).

The terms 'Before Axon' and 'After Axon' precisely capture the emphasis and organizational structure of our review. For a visual representation, we direct Reviewer-2 to Figure 1E, where a vertical gray line divides the PV neuron into two halves. The left half (panel "i") pertains to structures and processes best characterized as "Before Axon." While we acknowledge that some of these elements are associated with postsynaptic mechanisms, our term "Before Axon" also encompasses discussions on the laminar positioning of PV cells. This term aligns well with the comprehensive scope of our content and may more accurately convey the diversity of topics covered compared to the suggested term "postsynaptic mechanisms." Nevertheless, in response to this critique, we have added the following statement on page 7 to address and clarify this aspect: Traditional terms for "Before Axon" could be "Afferent Processes" or "Input Pathways," encompassing aspects such as synaptic integration. On the other hand, "After Axon" could be described as "Efferent Processes" or "Output Pathways," involving activities like neurotransmission. These terms align with conventional terminology used in neuroscience to distinguish between the incoming and outgoing signals of a neuron.

2 Similarly, 'faraway' versus 'local' inputs. I am not sure about the term 'faraway' as most readers would already understand that subcortical inputs to a particular cortical region are not local, and cortico-cortical inputs are by definition inputs from other cortical areas.

We defined "Local" and "Faraway" inputs separately on page 8, in section 3-02. Specifically:

Local - From Local Cells. The local group is made of cells in the same cortical column. …

Faraway - From Faraway Regions. Any axon projection originating outside the cortical column (in which our PV interneuron resides) is here denoted "faraway" or "remote". …

3 It is common for many reviews and articles to mix terms such as type, subtype, class, subclass. It would help to be consistent, e.g. PV+ neurons form a subpopulation of cortical GABAergic neurons. Within this subpopulation, there are several types, with similar features within each cortical area. In the hippocampus, there are several types of PV interneurons that also express somatostatin and/or NPY, which is different to the isocortical PV cells.

Reviewer-2 has astutely identified a critical issue in our work—namely, the tendency to conflate isocortical and allocortical PV interneurons. Moreover, there is an observed inconsistency wherein features confirmed solely in allocortical PV interneurons are at times erroneously attributed to isocortical PV interneurons.

In response to this insightful critique, we have refined the initial three lines of the

Introduction: Introduction This review centers on the examination of fast-spiking parvalbumin-expressing (PV+) interneurons within the neocortex. Acknowledging occasional limitations in direct neocortical

measurements, we have incorporated certain features observed in hippocampal PV+ interneurons (e.g., Reversed Rm gradient, section 3-06). We have assembled a list of interesting anatomical and physiological features that uniquely congregate in one cell type (PV+ interneuron). …

4 Despite the first sentence of the Introduction, it is not always clear if the review is about neocortical PV cells or all cortical PV cells (including the hippocampus). In most cases, the cortical area is specified, but there are several places where the specific region of cortex would help the reader assess the statement/evidence. There is a danger to generalise findings. For example, the main claim of a reversed Rm gradient (even mentioned in the abstract) is based on Norenberg et al 2010, which is from the dentate gyrus - clearly not the neocortex. The dentate gyrus is a highly specialized area and PV cells in other cortical areas may be different.

To address this critique, we inserted the following text into section 3-06 devoted to the reversed Rm gradient.

There is a risk associated with generalizing published findings. For instance, the primary assertion of a 'reversed Rm gradient' relies on the study by Norenberg et al. in 2010, conducted specifically in the dentate gyrus — a region notably distinct from the neocortex. The dentate gyrus represents a highly specialized area, and it's essential to recognize that the characteristics of PV cells in other cortical areas maydiffersignificantly.

5 Another claim is that PV neurons are the only type of myelinated interneuron. But many types of GABAergic neuron (including somatostatin-expressing neurons) in the hippocampus are myelinated, and many also have local axon and projection axons.

We acknowledge the reviewer's point regarding the need to avoid binary statements. In section 3-11, dedicated to the discussion of myelin, we have incorporated the following clarification:

"Among all cortical inhibitory GABAergic interneurons, only the PV+ subclass is reported to possess a myelinated axon (Stedehouder et al., 2017)."

It's important to note that this statement reflects the findings and novelty as presented by Stedehouder et al., 2017.

6 To avoid misleading the reader, it would help to (1) specify the exact sources of the observation(s), (2) avoid making binary statements (e.g. 'no other interneuron is myelinated' should be 'myelinated axons have only been observed in PV neurons in the neocortex' - other types of interneurons may well have myelinated axons and they just have not been detected yet).

We agree.

(1) We have explicitly identified the source of the observation as Stedehouder et al., 2017.

(2) It is crucial to underscore the unequivocal (binary) statement made by Stedehouder et al., 2017, as it serves as the focal point of Section 3-11.

(3) Additionally, we have included the following statement: "To date, myelinated axons have exclusively been observed in PV neurons. While other types of isocortical interneurons (SST, VIP) may potentially possess myelinated axons, they have not yet been detected."

7 Another example statement: "Information can neither enter nor leave the neocortex without the engagement of the fast PV+ mediated cortical inhibition" - evidence? One could use different terms depending on the amount of evidence or confidence ('we hypothesize', 'we speculate', 'we suggest' etc.). After careful consideration of the critique, we affirm the accuracy of our statement: 'Information can neither enter nor leave the neocortex without strong engagement of fast PV+ cell-mediated inhibition.' Our extensive exploration of cortical circuitry and processing consistently supports the notion that information flow in and out of the neocortex is contingent upon robust involvement of fast PV+ cell-mediated inhibition. This is exemplified by afferent information invoking feedforward inhibition through engagement with PV+ interneurons, and efferent information triggering feedback inhibition through similar interaction with PV+ interneurons.

We understand that the old highlights did not provide sufficient explanation of our statement, and it may have been confusing to readers. Therefore, in the revised highlights, we now state the following:

“This is exemplified by afferent information invoking feedforward inhibition through engagement with cortical PV+ interneurons, and efferent information triggering feedback inhibition through interaction with PV+ interneurons, before exiting neocortex”.

8 Somogyi P, Freund TF, Cowey A (1982) The axo-axonic interneuron in the cerebral cortex of the rat, cat and monkey. Neuroscience 7: 2577-2607. This is the first evidence of myelination of axo-axonic cell axons. We express our gratitude to Reviewer-2 for bringing attention to this article, an oversight on our part. This publication, which we regrettably missed, holds significance not only for unveiling the presence of myelin in PV+ chandelier cells but also for furnishing evidence that underscores the confinement of myelin to the primary axon-trunk (main axon) of PV+ cells. Notably, this study stands as one of the pioneering investigations shedding light on the exclusive target-specificity of these cells, specifically onto the axon initial segment of pyramidal neurons.

More specific comments:

9 Title: High-frequency oscillations, if defined in terms of the local field potential (LFP), emerge from the summed extracellular currents flowing across the membranes of all cells, not just the PV cells (eg see Buzsaki et al Nat Rev Neurosci. 2012;13:407-20).

We wholeheartedly concur. We carefully examined our test for statements that would challenge the established notion that the local field potential (LFP) arises from the cumulative extracellular currents flowing across the membranes of all cells.

To eliminate any potential ambiguity, we have included the following clarification on page 6:

“High-frequency oscillations, if defined in terms of the local field potential (LFP), emerge from the summed extracellular currents flowing across the membranes of all cells, not just the PV cells (Buzsaki et al., 2012). However, PV+ interneurons are the main component of the cortical circuitry that generates fast cortical oscillations (Cardin et al., 2009; Dubey et al., 2022; Sohal et al., 2009; Traub et al., 1996)”.

10 Alternative title: Physiological Features of Parvalbumin-Expressing GABAergic Neurons Contributing to High-Frequency Oscillations in the Cerebral Cortex / Neocortex.

We thank the reviewer for the title suggestion, and we accept this suggestion with a minor modification. Therefore, the new title is: Physiological Features of Parvalbumin-Expressing GABAergic Interneurons Contributing to HighFrequency Oscillations in the Cerebral Cortex

11 Public and media statement: Typo Careers. 'Physiology of' - physiological roles of? Corrected.

12 If this is a review for early-career scientists, one should briefly define GABA and define inhibition in general, as well as hyperpolarizing and shunting. See also recent paper on fast synaptic inhibition in vivo (Burman et al Neuron 2023 Active cortical networks promote shunting fast synaptic inhibition in vivo). Good point. In the second paragraph of Introduction, we now include the following definitions:

Gamma-aminobutyric acid (GABA) functions as the principal inhibitory neurotransmitter in the cerebral cortex. Upon binding to GABA-A receptors, GABA elicits either hyperpolarizing or shunting inhibition. Hyperpolarizing inhibition occurs when the equilibrium potential of the GABA-A receptor (ECl) is more negative than the neuron's membrane potential (Vm), resulting in typical inhibitory postsynaptic potentials (IPSPs). Shunting inhibition, on the other hand, takes place when ECl equals Vm (Burman et al., 2023). Note that transmembrane current (IGABA) is the product of receptor conductance (gGABA) and the driving force (Vm – ECl); [IGABA = gGABA * (Vm – ECl)]. In instances where Vm equals ECl, an increase in gGABA-A (GABAergic input) may transpire without an overt change in the postsynaptic neuron's membrane potential, as the driving force for the GABA-A current is zero (Vm – ECl = 0). Both classes of GABAergic inputs, hyperpolarizing and shunting, exert potent inhibition (Burman et al., 2023). Page 5 of 9 We thank the reviewer for bringing Burman et al (2023) to our attention.

13 Highlights: The term 'outpace' is unclear, as are 'competing networks'.

It would be impractical to expect the authors to elucidate every term already in the article highlights. From our perspective, the statement 'PV+ interneurons use fast spiking to outpace other excitable cells in the cortex and sculpt the ongoing electrical rhythm' is lucid. This implies that PV+ interneurons can generate spikes more rapidly and at a higher frequency than the 'competing network' comprised of cortical pyramidal neurons. In our current conceptualization, we envision the cerebral cortex as consisting of two competing cortical networks: one constituted by slow and numerous pyramidal cells releasing glutamate, and the other network composed of fast yet scarce interneurons releasing GABA.

Per reviewer’s critique, we now include the following statement into the Highlights:

“We envision the cerebral cortex as consisting of two competing cortical networks: one constituted by slow and numerous pyramidal cells releasing glutamate, and the other network composed of fast yet scarce interneurons releasing GABA”.

14Overwhelm=overcome? Corrected!

15 PV+ interneurons inhibit other excitable (glutamatergic, or excitatory?) cells within short time windows during the ongoing electrical rhythm - all neurons are excitable. Corrected! The new text reads: [i] Fast spiking ability that allows PV+ interneurons to outpace other cortical neurons (pyramidal neurons).

16 'Strategic positioning of the PV+ inhibitory synapses'. 'Strategic' suggests there was a strategy, rather it is evolution/development. The term 'Strategic' in the context of 'Strategic positioning of the PV+ inhibitory synapses' does not imply a conscious strategy but rather emphasizes the outcome of evolutionary and developmental processes. Through evolutionary pressures, PV+ synapses have adapted

and “discovered” an effective approach, akin to a strategy, for inhibiting spikes in pyramidal cells. This strategy is inherently simple – prioritize the axon initial segment over other parts of pyramidal neurons. Reviewer-2 has pointed out an article we initially overlooked: Somogyi P, Freund TF, Cowey A (1982). Interestingly, in this article, the authors state in their abstract: 'Axo-axonic cells are uniquely endowed with the means of simultaneously influencing the action potential at the site of origin in groups of pyramidal cells. This strategic location may enable them to synchronize the activity of pyramidal neurons, either through inhibitory gating or by altering the threshold of pyramidal cells to certain inputs.' It is interesting that Reviewer-2 directs us to Somogyi et al., 1982, where the term 'strategic' is used to describe the advantageous positioning of axoaxonic cells, aligning with our understanding of the PV+ inhibitory synapses.

17 Are spikes obliterated or just that the membrane potential is hyperpolarized or there is shunting? Evidence for a 100% success rate? We agree. The expression: “100% success rate” was inappropriately strong. We amended this text in the following manner: “… to robustly obliterate spiking in cortical pyramidal cells, or paradoxically excite cortical pyramidal neurons, with a high success rate. “

18 Abstract: 'It would be useful to carry out [further] physiological recordings [of] PV+ cells selectively. See existing published studies on identified PV cells and cognition e.g. Lagler et al Neuron 2016 Divisions of Identified Parvalbumin-Expressing Basket Cells during Working Memory-Guided Decision Making. Again, we thank the reviewer for bringing this study to our attention. This resulted in several instances of new text: 18-1 The same pyramidal cell (PC) participates meaningfully in the function of many different neuronal ensembles (Desimone et al., 1984; Legenstein and Maass, 2017; Wilson and McNaughton, 1993). The same is true for inhibitory PV cells (Lagler et al., 2016). 18-2 Firing patterns of PV+ basket cells might support and temporally organize neuronal ensembles of the neocortex during various brain states and tasks, including non-REM sleep and working memory-guided decision making (Funk et al., 2017; Lagler et al., 2016). 18-3 Single pulse stimulation of the hippocampal CA1 or subiculum region induce an excitatory response in 70% of recorded interneurons in the prelimbic and medial-orbital areas of the rat prefrontal cortex (Tierney et al., 2004). Hippocampal projections to prefrontal cortex carry spatial information, and support working memory (Ciocchi et al., 2015; Spellman et al., 2015), where the successful encoding of hippocampal cues appears to be mediated by oscillation-synchrony between hippocampus and PFC (Hartwich et al., 2009; Spellman et al., 2015).

19 "The overwhelming majority of PV-to-PV electrical synapses are located on cell body and near the cell body." - meaning between a cell body and a dendrite or 2 cell bodies?

We understand the issue brought up by reviewer-2. To address this critique, we produced the following change: The overwhelming majority of PV-to-PV electrical synapses are located on the cell body and near the cell body, on the most proximal segments of dendritic branches (Galarreta and Hestrin, 1999; Gibson et al., 1999; Tamas et al., 2000).

20 Introduction: 'Really fast and mighty strong' - not clear terms, and in relation to what other cell types and timescales? Claim of 'never before included in a PV interneuron review article' - this is a potentially misleading statement, perhaps unnecessary. We agree. That statement is potentially misleading and perhaps unnecessary. It has been removed from the Introduction, and replaced by the following syntax: … properties which did not receive much attention in PV+-interneuron review-articles …

21 Type-2 firing is not defined. What is type-1 firing? There are two types of threshold behavior for periodically firing neurons, which show continuous (Type-1) or discontinuous (Type-2) f–I curves. Tateno et al (2004) have shown that in the cortex, regular-spiking and fast-spiking neuronal types have Type-1 and Type-2 thresholds, respectively. This endows RS neurons and FS neurons with fundamentally different: [a] spike rate encoding, [b] noise sensitivity, [c] subthreshold, and [d] synchronization properties. Thus RS neurons and FS neurons are expected to serve very different roles in the dynamics of the cortical network. Neurons and dynamical models of spike generation display two different types of threshold behavior, with steady current injection: Type-1 [the firing frequency vs. current (f–I) relationship is continuous at threshold) and Type-2 (discontinuous f–I)]. Regular spiking (RS) pyramidal neurons show a Type-1 threshold, consistent with their well-known ability to fire regularly at very low frequencies. In fast-spiking (FS) inhibitory interneurons, although regular firing is supported over a wide range of frequencies, there is a clear discontinuity in their f–I relationship at threshold (Type-2). FS neurons are unable to support maintained periodic firing below a critical frequency fc, in the range of 10 to 30 Hz. Very close to threshold, FS cells switch irregularly between bursts of periodic firing and subthreshold oscillations. These characteristics mean that the dynamics of RS neurons are well suited to encoding inputs into low-frequency firing rates, whereas the dynamics of FS neurons are suited to maintaining and quickly synchronizing to gamma and higher-frequency input.

22 Minor: figures are not cited in order. We faced the choice between increasing the number of figures, so they can be cut into pieces and then cited in a perfect order or keeping the current number of figures to 5. Faced with the decision of either increasing the number of figures to facilitate segmentation for optimal citation order or maintaining the current count at 5 figures, we believe that the latter option is more elegant.

23 'Unnecessary spikes' and 'sparse coding' - need strong definitions here as this is subjective and dependent on behavioural state, model, anesthesia, and brain region, and also recording/imaging technique. E.g. 'sparse coding' is often referred in models where all but one whisker are trimmed, focusing on mouse somatosensory cortex. In this paragraph, again the brain region ("sensory" cortex or other cortical areas should be specified as may not apply to all cortical areas). “Unnecessary spikes” are defined by our text and Figure 1AB. Page 8 of 9 “Sparse coding” is defined in Pala and Petersen, 2015. The brain regions in question are somatosensory cortex and motor cortex. We revised the manuscript text in the following manner: “In somatosensory and motor cortices, a few interneuron spikes prevent many unnecessary pyramidal cell spikes (Fries et al., 2007), promoting sparse coding (Pala and Petersen, 2015)”.

24 'Cumbersome baseline firing' - again this term is subjective, as brain operations are not normally considered cumbersome, given energy demands. We appreciate the reviewer's feedback and understand the concern about the subjective nature of the term 'cumbersome baseline firing.' To provide clarity, we define this term in the context of our study: 'cumbersome baseline firing' refers to an overtly strong baseline firing that has two detrimental effects. First, it hinders the accuracy of sensory processing (Fig. 1AB). Second, it leads to unnecessary energy expenditure. To illustrate the potential impact, consider an animal cortex with only 10 million neurons, each neuron firing 3 unnecessary spikes per second. Over a wake cycle of 16 hours, this would result in 1.7 trillion wasteful spikes. We argue that such a substantial number of wasteful spikes significantly contributes to energy consumption.

25 "Suppression of the baseline firing" paragraph - claim of 200um region, specify if this is a diameter or width of a cortical area, and which cortical region it refers. In the revised manuscript, we specify cortical regions and 200 µm radius in a coronal section. The revised text is worded in the following manner: In

somatosensory or frontal rodent cortex, through a widespread and non-specific inhibition of nearly every pyramidal neuron within a 200 µm radius (Karnani et al., 2014; Packer and Yuste, 2011), PV+ cells diminish the activity of PCs without altering their: tuning, sensitivity, class or object preference, and other specific response properties (Atallah et al., 2012; Spanne and Jorntell, 2015; Trachtenberg, 2015).

26 "Pyramidal neuron network dynamic range" paragraph - specify which brain area and species these data were obtained. Now specified: in the mouse barrel cortex

27 Neuronal ensembles - see also neuronal assemblies. The use of these terms is often brain-region dependent and behavior-dependent. Suggest giving examples or specifying brain regions referring to. We appreciate the reviewer's consideration and acknowledge that the terms 'neuronal ensembles' and 'neuronal assemblies' may exhibit variability across brain regions and behaviors. In the context of our discussion, which centers on the most general principles, we agree that minute differences between neuronal assemblies in various brain regions are both unknown to us and beyond the scope of the current article.

28 "Pyramidal cells (PCs) that respond to animal-like objects appearing in the perceptual space, should not respond when plant-like objects appear" - evidence? Is this statement a speculation? 'Plant-like' object cells and animal-like object cells - If no evidence, state these as examples or hypotheses, as the cited papers seem to not relate to them. In the section titled: "Suppression of the out-of-context cells", we now include the following three disclaimers. 28-1 Here we posit that PV cells play an important role in determining which PC is recruited into a functional neuronal ensemble, … 28-2 We hypothesize that pyramidal cells (PCs) that respond to animal-like objects appearing in the perceptual space, should not respond when plant-like objects appear. 28-3 We hypothesize that these long-lasting cell-body depolarizations arise from clustered glutamatergic inputs (inputs on one dendritic branch) which trigger dendritic plateau potentials …

29 Gamma oscillations: Further insight into different types of gamma oscillations and coupling (relevant to PV cells) see Lasztóczi B, Klausberger T. Distinct gamma oscillations in the distal dendritic fields of the dentate gyrus and the CA1 area of mouse hippocampus. Brain Struct Funct. 2017;222(7):3355-3365. In section 2 (Fast Electrical Oscillation - Gamma Band) we now cite Lasztoczi and Klausberger (2017). The revised text reads as follows: Neuronal gamma-band synchronization (30-80 Hz) appears to be a fundamental mode of neuronal activity (Fries et al., 2007; Jefferys et al., 1996; Lasztoczi and Klausberger, 2017; Salkoff et al., 2015).

30 Box 1, typo glutamate receptors Corrected in Box 1, and Section 3-05, and Graphical Abstract.

31 Box 2, GABA 'release onto' rather than 'delivery on'? Corrected in Box 2, and Section 3-14, 3-15, and Graphical Abstract.

Sincerely, The authors

Accept Letter

Dear Associate Professor Antic,

Thank you for submitting your manuscript to Current Research in Neurobiology.

I am pleased to inform you that your manuscript has been accepted for publication.

My comments, and any reviewer comments, are below.

Your accepted manuscript will now be transferred to our production department. We will create a proof which you will be asked to check, and you will also be asked to complete a number of online forms required for publication. If we need additional information from you during the production process, we will contact you directly.

We appreciate and value your contribution to Current Research in Neurobiology. We regularly invite authors of recently published manuscript to participate in the peer review process. If you were not already part of the journal's reviewer pool, you have now been added to it. We look forward to your continued participation in our journal, and we hope you will consider us again for future submissions.

CRNEUR aims to be a unique, community-led journal, as highlighted in the Editorial Introduction. As part of this vision, we will be regularly seeking input from the scientific community and encourage you and your co-authors to take the survey.

We would also like to invite you to take part in our CRNEUR Author Question & Answer (Q&A), which could get published alongside your article and help to promote it. We suspect you might have an interesting story of perseverance or team work that was required for the research study to complete, or a diversity of perspectives that you might share, as a way of inspiring others about neuroscience.

Kind regards,

Abhishek Banerjee
Associate Editor
Current Research in Neurobiology

-------- *End of Review Comments* --------
